# Complex post-traumatic stress disorder in refugees, asylum seekers, and internally displaced people: a systematic review and meta-analysis

**DOI:** 10.1080/20008066.2026.2735202

**Published:** 2026-10-20

**Authors:** Mamaru Melkam, Cheng Xu, Fiona Maccallum, Lena Oestreich

**Affiliations:** a School of Psychology, The University of Queensland, Brisbane, Australia; b Department of Psychiatry, College of Medicine and Health Science, University of Gondar, Gondar, Ethiopia; c School of Psychology and Cognitive Science, East China Normal University, Shanghai, People’s Republic of China; d Australian Institute for Bioengineering and Nanotechnology, The University of Queensland, Brisbane, Australia, Australia

**Keywords:** PTSD, CPTSD, refugees, IDPs, asylum seekers, conflict-affected areas, TEPT, TEPT-C, refugiados, personas que buscan asilo, áreas afectadas por conflicto, personas desplazadas internamente

## Abstract

**Background:** Forcibly displaced populations, including refugees, asylum seekers, and internally displaced persons, are frequently exposed to severe, prolonged and repeated trauma from which escape is difficult or impossible. Such exposures increase the risk for complex post-traumatic stress disorder (CPTSD), defined by core PTSD symptoms alongside disturbances in self-organisation (DSO), including affective dysregulation, negative self-concept, and relational difficulties. However, the prevalence of CPTSD in forcibly displaced populations has not yet been quantitatively synthesised, and the added value of the CPTSD construct over PTSD in this population has not been examined.

**Methods:** This systematic review and meta-analysis searched five databases from inception to January 2026. A random-effects meta-analysis was conducted to estimate pooled CPTSD prevalence, with pre-specified subgroup analyses, meta-regression, and sensitivity analyses.

**Results:** Twenty studies met inclusion criteria, representing 17 independent samples (*n* = 12,504). Reported CPTSD prevalence varied from 3.0% to 74.6%. The random-effects pooled prevalence, retained as a descriptive summary of the included studies, was 20.9% (95% CI [12.6%, 32.6%]), the 95% prediction interval was [1.7%, 80.3%]. Between-study heterogeneity was very high (*I²* = 98.3%). Recruitment context was strongly associated with prevalence: estimates averaged 10.6% in community-based samples and 55.2% in treatment-seeking samples. Asylum seekers exhibited markedly higher prevalence than refugees. Across four studies, post-migration living difficulties, including legal insecurity, poverty, and marginalisation, were more strongly associated with DSO symptoms than with core PTSD symptoms, suggesting that ongoing structural adversity actively perpetuates complex trauma psychopathology beyond pre-migration trauma alone.

**Conclusion:** Available evidence indicates marked variation in CPTSD prevalence across forcibly displaced populations rather than a single generalisable prevalence rate. Recruitment context accounts for part of this variation, but the limited and geographically uneven evidence base prevents global population-level inference. Future studies should use representative sampling and consistently report displacement, trauma, and post-migration characteristics to permit more informative explanations of between-population variation.

## HIGHLIGHTS

- Reported complex post-traumatic stress disorder (CPTSD) prevalence varied markedly across forcibly displaced populations and settings.
- Rates of CPTSD were especially high among people seeking treatment and among asylum seekers, suggesting that ongoing uncertainty and adversity may intensify psychological distress.
- Ongoing challenges after displacement, including poverty, legal insecurity, and social exclusion, were more strongly linked to complex trauma symptoms than past trauma alone, underscoring the need for both psychological care and social support.

## Abstract (es)

**Antecedentes:** Las poblaciones desplazadas por la fuerza, incluyendo los refugiados, personas que buscan asilo, y las personas desplazadas internamente, están frecuentemente expuestas a traumas graves, prolongados y repetidos de los cuales escapar es difícil o imposible. Tales exposiciones aumentan el riesgo de trastorno de estrés traumático complejo (TEPT-C), definido por los síntomas TEPT centrales junto con las perturbaciones en la autoorganización (DSO en su sigla en inglés), incluyendo desregulación emocional, autoconcepto negativo, y dificultades relacionales. Sin embargo, la prevalencia del TEPT-C en las poblaciones desplazadas por la fuerza no ha sido sintetizada de forma cuantitativa, y el valor agregado del constructo TEPT-C sobre el TEPT en esta población no ha sido examinado.

**Métodos:** Esta revisión sistemática y metaanálisis consultó en cinco bases de datos desde su inicio hasta enero de 2026. Se realizó un metaanálisis de efectos aleatorios para estimar la prevalencia combinada, con análisis de subgrupos preespecificados, meta regresión y análisis de sensibilidad.

**Resultados:** Veinte estudios cumplieron con los criterios de inclusión, representando 17 muestras independientes (*n* = 12.504). La prevalencia reportada de TEPT-C varió desde 3.0% a 74.6%. La prevalencia combinada de efectos aleatorios, mantenida como un resumen descriptivo de los estudios incluidos, fue de 20.9% (IC 95% [12.6%, 32.6%]), el intervalo de predicción del 95% fue [1.7%, 80.3%]. La heterogeneidad entre los estudios fue muy alta (I² = 98.3%). El contexto de reclutamiento se asoció fuertemente con la prevalencia: estimaciones promediaron 10.6% en muestras comunitarias y 55.2% en muestras de quienes buscaban tratamiento. Las personas que buscaban asilo mostraron una prevalencia marcadamente mayor que la de los refugiados. A través de cuatro estudios, las dificultades de vida posmigración, incluyendo inseguridad legal, pobreza y marginalización, se asociaron más fuertemente con los síntomas de DSO que con los síntomas centrales de TEPT, lo que sugiere que la adversidad estructural permanente perpetúa activamente la psicopatología de trauma complejo más allá del trauma pre-migración por sí solo.

**Conclusiones:** La evidencia disponible indica una marcada variación en la prevalencia del TEPT-C a lo largo de poblaciones desplazadas por la fuerza más que una tasa de prevalencia única y generalizable. El contexto de reclutamiento explica parte de esta variación, pero base de la evidencia limitada y geográficamente desigual, impide realizar una inferencia a nivel de la población global. Las investigaciones futuras deberían usar muestras representativas y reportar de forma consistente las características del desplazamiento, trauma, y posmigración para permitir explicaciones más informativas de la variación entre las poblaciones.

## 1. Introduction

Complex post-traumatic stress disorder (CPTSD) was formally recognised in the International Classification of Diseases (ICD-11) as a trauma-related disorder distinct from post-traumatic stress disorder (PTSD) (World Health Organization, 2019/2021). PTSD is characterised by re-experiencing of the traumatic event in the present, avoidance of trauma reminders, and a persistent sense of current threat (Brewin et al., 2017). CPTSD encompasses these core symptoms alongside disturbances in self-organisation (DSO), comprising affective dysregulation, negative self-concept, and relational difficulties, reflecting the broader and more enduring consequences of sustained, repeated, or interpersonal trauma, particularly in contexts involving captivity, coercion, or dependency (Cloitre et al., 2013; Maercker et al., 2013).

Refugees, asylum seekers, and internally displaced persons (IDPs) represent populations at particular risk for CPTSD given the severity and chronicity of their trauma exposures. Refugees are individuals recognised under international law as requiring protection after fleeing persecution across an international border (Gerasymenko et al., 2022). Asylum seekers are individuals who have fled their home country and are seeking international protection but whose claims for refugee status have not yet been legally determined (Douglas et al., 2019). In contrast, IDPs are individuals forcibly displaced within their country of origin by conflict, violence, human rights violations, or disaster (Gerasymenko et al., 2022). As of mid-2025, more than 117 million people were forcibly displaced globally, including approximately 42.5 million refugees, 8.4 million asylum seekers, and 67.8 million IDPs (United Nations High Commissioner for Refugees 2025). Given the scale of global displacement, understanding the mental health consequences of these experiences represents a pressing public health priority.

Forcibly displaced populations are exposed to severe and cumulative trauma across multiple displacement phases. Pre-migration experiences commonly include war, torture, persecution, and systematic violations of basic human rights, including the denial of safe housing and access to healthcare (Mellor et al., 2021). Post-displacement stressors such as legal uncertainty, family separation, poverty, unemployment, stigma, and exploitation compound prior trauma exposure (de Silva et al., 2021; Hecker et al., 2018). Ongoing concern for family in conflict zones, traumatic grief, and survivor's guilt may further intensify psychological burden (Cloitre, 2022; de Silva et al., 2021). This accumulation of interpersonal and chronic stressors maps closely onto the etiological conditions theorised to precipitate CPTSD (Tay et al., 2015).

Distinguishing CPTSD from PTSD is not merely a nosological refinement. It has particular relevance for forcibly displaced populations. ICD-11 conceptualises CPTSD as the characteristic outcome of prolonged, repeated, and interpersonal trauma from which escape is difficult or impossible (Cloitre et al., 2013; Maercker et al., 2013), an exposure profile that closely matches persecution, torture, detention, and protracted displacement. Critically, the DSO symptoms that define CPTSD (affective dysregulation, negative self-concept, and disturbed relationships) are associated with greater functional impairment and psychiatric comorbidity than core PTSD symptoms alone (Cloitre et al., 2019; Karatzias et al., 2019a), and they map onto the difficulties, including shame, guilt, mistrust, and disrupted relationships, that are most likely to impede integration and help-seeking after resettlement (Gerasymenko et al., 2022; Tay et al., 2015). Prevalence research restricted to PTSD therefore risks underestimating and mischaracterising the mental health burden of forced displacement, and provides little guidance on whether the additional treatment targets implied by DSO are relevant at population scale. Establishing how frequently ICD-11 CPTSD occurs in displaced populations, and how its prevalence varies across settings, is thus a prerequisite both for epidemiological burden estimation and for treatment research asking whether displaced people with CPTSD have needs beyond those addressed by PTSD-focused care.

Prevalence estimates of CPTSD vary substantially across populations and levels of trauma exposure. In a population-based study in the United States (US), CPTSD prevalence was estimated at 3.8% (Cloitre et al., 2019). In the United Kingdom (UK), cross-sectional studies reported a prevalence of 12.9% among trauma-exposed individuals (Karatzias et al., 2019b). In forcibly displaced populations, estimates range far more widely. For instance, a study of African refugees in Italy reported a CPTSD prevalence of 30% (Barbieri et al., 2019), while a study of asylum seekers in a humanitarian setting in Africa reported a prevalence of 74.6% (Barbieri et al., 2023). In contrast, a study of West Papuan refugees reported prevalence rates of 9.3% for CPTSD and 12.1% for PTSD (Silove et al., 2018). This heterogeneity in CPTSD presentations among forcibly displaced populations likely reflects the interplay of sociodemographic factors, such as younger age, female gender, low educational attainment, and social isolation (Hyland et al., 2021; Rawers et al., 2024), trauma-related variables, including interpersonal violence, detention and bereavement (Andisha et al., 2023; Barbieri et al., 2019; Karstoft et al., 2024), as well as post-migration stressors, such as legal precarity, poverty, and marginalisation (de Silva et al., 2021; Hecker et al., 2018).

Despite a growing body of research examining CPTSD in forcibly displaced populations, the extent and sources of variation in reported prevalence have not been systematically synthesised. Estimates are likely to differ across recruitment settings, displacement groups, conflict and resettlement contexts, and measurement approaches. The primary aim of this review was therefore to characterise the distribution and variability of CPTSD prevalence estimates among forcibly displaced adults and to examine whether available study-level characteristics accounted for this variation. The fact that reported prevalence estimates vary so widely across studies, ranging from 3% to over 70% (Barbieri et al., 2023; Hecker et al., 2018; Silove et al., 2017; Tay et al., 2015; Vang et al., 2020), complicates burden estimation and impedes evidence-based service planning. The present study addresses this gap by systematically reviewing the literature on CPTSD among refugees, asylum seekers, and IDPs, and quantitatively synthesising prevalence estimates as a descriptive summary of the included studies. A secondary aim was to synthesise evidence on demographic, clinical, trauma-related, and post-migration correlates of CPTSD.

## 2. Methods

### 2.1. Study design

This systematic review and meta-analysis was conducted in accordance with the Preferred Reporting Items for Systematic Review and Meta-Analysis (PRISMA) 2020 guidelines (see Supplementary Table 1) (Page et al., 2021). The review protocol was preregistered on the Open Science Framework (Melkam et al., 2026).

### 2.2. Search strategy

A systematic literature search was conducted across MEDLINE (via PubMed), Embase, CINAHL, PsycINFO, and the Cochrane Library. The search was conducted from the inception of each database through 28 January 2026. The search strategy was developed in consultation with a librarian at the University of Queensland. Medical Subject Headings (MeSH), keywords, abbreviations, and acronyms of CPTSD and displacement were included. Search strings were adapted for each database and boolean operators, truncation symbols, and field tags were applied as appropriate. The core search concept combined terms for (i) CPTSD and (ii) displacement:

- “Complex PTSD” OR “ICD-11 complex PTSD” OR “cptsd” OR “complex post-traumatic stress disorder” OR “complex post-traumatic stress disorder” OR “C-PTSD” OR “complex trauma*” OR “developmental trauma*” OR “repeated trauma*” OR “chronic trauma*” OR “traumatic event*”
- AND “internally displaced” OR “internal displacement*” OR “IDP*” OR “refugee*” OR “asylum seeker*” OR “forcibly displaced*” OR “resettlement*” “forced migrant*” OR “displaced population*” OR “conflict-affected” OR “war-affected” OR “humanitarian crisis*” OR “emergency-affected” OR “emergency setting*” OR “civil war*” OR “people affected by war*” OR “war survivor*” OR “war exposure*” OR “traumatized refugee*” OR “post migration*” OR “mass casualty*” OR “post-migration*”.

Reference lists of all included studies were also screened to identify additional eligible studies.

### 2.3. Eligibility criteria

Studies were eligible for inclusion if they met the following criteria: (i) the study sample consisted of forcibly displaced adults (≥18 years), including refugees, asylum seekers, and IDPs, as defined by international legal frameworks (i.e. individuals who have been forcibly displaced from their homes due to conflict, persecution, or violence); (ii) the study reported the prevalence of ICD-11 CPTSD, either as a point prevalence or as a diagnostic proportion; (iii) CPTSD was assessed using a validated ICD-11-compatible instrument, such as the International Trauma Questionnaire (ITQ) (Cloitre et al., 2018), a brief self-report measure developed specifically to operationalise ICD-11 PTSD and CPTSD criteria across six symptom clusters, or other validated instruments with items explicitly mapped onto the ICD-11 CPTSD diagnostic criteria; (iv) the study employed an observational design (e.g. cross-sectional, cohort, or epidemiological survey); and (v) the study was published in a peer-reviewed journal in English.

Studies were excluded if (i) the sample did not consist of forcibly displaced populations (e.g. general community residents affected by war, military veterans, or prisoners of war, who differ fundamentally from displaced populations in their exposure context and post-displacement circumstances); (ii) the study assessed PTSD without reporting CPTSD prevalence; (iii) the study employed an instrument which cannot yield valid ICD-11 CPTSD prevalence estimates; (iv) the study focused exclusively on children or adolescents (<18 years); (v) the study was a Randomized Controlled Trial (RCT), review, case report, commentary, editorial, or qualitative study; or (vi) the study did not report sufficient data to calculate a prevalence estimate.

### 2.4. Study selection and data extraction

All identified records were imported into Covidence for screening (Hupe, 2025). Title and abstract screening was independently conducted by two reviewers (MM and CX), followed by full-text review of potentially eligible studies. At each stage, disagreements were resolved through discussion, and where consensus could not be reached, a third reviewer (LO) was consulted. The study selection process is summarised in the PRISMA flow diagram (Figure 1).

**Figure 1.**
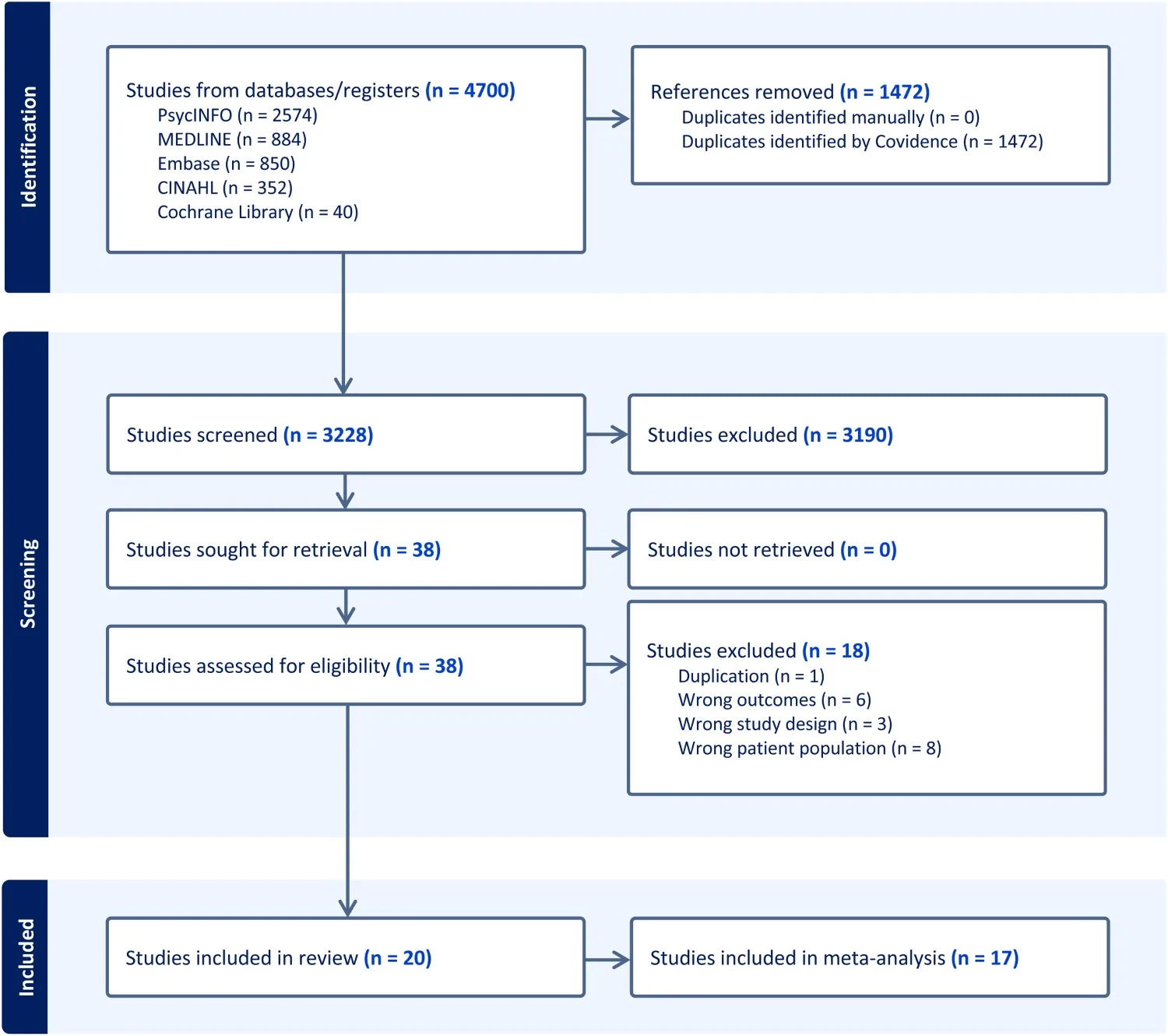
PRISMA flow diagram of study selection. Flow diagram illustrating the identification, screening, eligibility assessment, and inclusion of studies in the systematic review. A total of 4,700 records were identified through database searches (PsycINFO, MEDLINE, Embase, CINAHL, and Cochrane Library). After removal of 1,472 duplicate records, 3,228 studies were screened by title and abstract. Of these, 38 studies were retrieved for full-text assessment, and 18 studies were excluded for the following reasons: duplication (*n* = 1), wrong outcomes (*n* = 6), wrong study design (*n* = 2), wrong patient population (*n* = 8), and wrong route of administration (*n* = 1). In total, 20 studies met inclusion criteria and were included in the systematic review.

Data were extracted independently by two reviewers (MM and LO) using a standardised, pre-piloted extraction form developed in Excel. Discrepancy was again resolved through discussion. The following information was extracted from each study: first author and year of publication, study aims, study design, country or region of displacement origin, country of resettlement, sample characteristics (sample size, age [mean ± *SD* or median with interquartile range (ITQ), as reported], sex distribution, time since displacement), sample type (refugees, asylum seekers, IDPs, or mixed), and recruitment context (treatment-seeking vs. community-based). The CPTSD assessment instrument was recorded with explicit distinction between studies that used the ITQ directly and those that used other instruments with items mapped onto ICD-11 CPTSD criteria. CPTSD prevalence data were extracted as the number of cases and total sample size, or as a reported proportion. Risk factors examined, covariates adjusted for, statistical analyses, and key findings were also recorded. Where multiple publications reported data from the same or overlapping participant samples, only the study with the larger sample size was retained for the meta-analysis, to avoid non-independence of effect sizes.

### 2.5. Quality assessment

Methodological quality of included studies was assessed using the Joanna Briggs Institute (JBI) Critical Appraisal Checklist for Prevalence Studies (Munn et al., 2014). Two reviewers (MM and LO) independently appraised each study, with disagreements resolved through discussion. In addition, because the validity of the CPTSD instrument in the language and population studied is critical to the interpretability of prevalence estimates, JBI item 6 (‘Were valid methods used for the identification of the condition?’) was expanded into a structured appraisal of instrument validity for every included study (informed by the approach of Hoppen et al., 2021) and adapted to self-report instruments (Hoppen et al., 2021). For each study we recorded (i) the instrument and language(s) of administration, (ii) whether translation and/or cultural adaptation procedures were reported, (iii) whether the instrument had been validated in the language(s) administered, (iv) whether it had been validated in a forcibly displaced population, and (v) the psychometric evidence reported in the study sample (Supplementary Table 2B). Item 6 was scored 1 where the instrument, in the language administered, had been validated in a forcibly displaced population, and 0 otherwise; this binary rating defined the instrument-validation subgroups used in the meta-analysis.

### 2.6. Data synthesis and statistical analysis

All statistical analyses were performed in R version 4.5.1 (RStudio Team, 2023), using the *meta* and *metafor* packages. For the primary analysis, the pooled prevalence of ICD-11 CPTSD was estimated using a random-effects model. Individual study proportions were transformed using the logit transformation prior to pooling, as this approach provides appropriate confidence intervals for proportions. Study-specific 95% confidence intervals were calculated using the Clopper–Pearson exact method and results are presented as back-transformed pooled prevalence estimates with 95% confidence intervals. Heterogeneity was quantified using the *I²* statistic, which describes the proportion of observed variability attributable to between-study heterogeneity, and *τ²*, which provides an absolute measure of between-study variance on the logit scale. The significance of heterogeneity was assessed using Cochran’s *Q* test. A 95% prediction interval was also calculated, providing a more clinically interpretable measure of heterogeneity by estimating the range of prevalence values within which the true prevalence of a future comparable study would be expected to fall. Because the included studies sampled heterogeneous populations rather than a single underlying global population, the random-effects mean was conceptualised as the average of the distribution of study-specific prevalence parameters represented in the available literature. It was not interpreted as the prevalence of CPTSD among all forcibly displaced adults globally.

To this end, pre-specified subgroup analyses were conducted to explore potential sources of heterogeneity using mixed-effects models (random effects within subgroups, fixed effects for the between-subgroup comparisons). The following categorical moderators were examined: (1) CPTSD measurement approach (ITQ vs. other ICD-11-mapped instruments); (2) recruitment context (treatment-seeking vs. community-based samples); (3) displacement subgroup (refugees vs. asylum seekers vs. IDPs vs. mixed samples); (4) income classification of the country of resettlement, based on the World Bank 2024/2025 classification (World Bank, 2025); and (5) instrument validation status in the language and population studied. Meta-regression was used to examine the associations between study-level characteristics and CPTSD prevalence, where sufficient data were available. Planned moderators included mean time since displacement, mean age, and proportion of female participants.

Two pre-specified sensitivity analyses were conducted to assess the robustness of the pooled estimate. First, a leave-one-out analysis was performed, sequentially removing each study to identify disproportionate influence on the pooled estimate. Second, analyses were stratified by JBI quality rating to assess whether methodological quality influenced the pooled prevalence. Publication bias was assessed visually by funnel plot inspection of logit-transformed study proportions against their standard errors, and formally tested using Egger’s and Peters’ tests.

## 3. Results

### 3.1. Study selection

A total of 4,700 articles were identified through database searches (MEDLINE = 884; Cochrane Library = 40; Embase = 850; CINAHL = 352; PsycINFO = 2,574). After removal of 1,472 duplicates, 3,228 records remained for screening. A total of 20 studies met the eligibility criteria and were included in the systematic review (Andisha et al., 2023; Bachem et al., 2024; Barbieri et al., 2019; 2023; Benoni et al., 2025; Frost et al., 2019; Hecker et al., 2018; Hyland et al., 2018; Ipekci, 2023; Jowett et al., 2021; Karstoft et al., 2024; 2025; Liddell et al., 2019; McGinty et al., 2023; Rawers et al., 2024; Silove et al., 2017; Tay et al., 2015; 2018; Vallières et al., 2018; Vang et al., 2020). The systematic search and screening process is summarised in the PRISMA flow diagram (Figure 1). Inter-rater agreement during title and abstract screening was high (observed agreement = 97.8%), with a Cohen’s κ of 0.45, indicating moderate agreement. The modest κ reflects the substantial class imbalance inherent in large-scale screening, where the majority of records are excluded. Three pairs of studies drew from overlapping participant samples: Hyland et al. (2018) and Vallières et al. (2018) reported on the same sample of treatment-seeking Syrian refugees in Lebanon. Karstoft et al. (2024, 2025) analysed the same cohort of Ukrainian refugees in Denmark, and Silove et al. (2017) and Tay et al. (2015) used the same sample of West Papuan refugees in Papua New Guinea. Vallières et al. (2018), Karstoft et al. (2024) and Tay et al. (2015) were retained for the meta-analysis on the basis of larger sample size.

### 3.2. Systematic review of included studies

#### 3.2.1. Study characteristics

The characteristics of all included studies are summarised in Table 1. All studies employed a cross-sectional design, with the exception being Karstoft et al. (2025), which used a prospective two-wave cohort design. Studies were published between 2015 and 2025, with the majority (*k* = 14) published from 2019 onwards, reflecting the relatively recent formalisation of the ICD-11 CPTSD diagnosis.

**Table 1.** Data extracted from the included articles on CPTSD among refugees, asylum seekers, and IDPs (*n* = 20).

Authors/ year	Aims	Study type	Displacement origin	Resettlement Country	Study sample	Sample size	CPTSD Prevalence	Age (mean ± *SD*)	Sex	Time since displacement (mean ± *SD*)	CPTSD tool	Risk factors examined	Covariates	Analyses	Result	Finding
Andisha et al. (2023)	Translate and culturally adapt the ITQ for use in Dari; assess construct validity and composite reliability of ICD-11 PTSD and CPTSD	Cross-sectional	Afghanistan (50.1%), Iran (46.9%), Pakistan (3.0%)	Austria	Asylum seekers (50.1%); Refugees (49.9%); mixed general and treatment-seeking	305	20.7%	31.38 ± 11.05	M = 202 (66.2%); F = 103 (33.8%)	Asylum seekers: M = 3.88y (*SD* = 1.08); refugees: M = 5.99y (*SD* = 2.02)	ITQ (Dari version)	Sex, age, pre-migration traumas, post-migration stressor, psychological distress, anxiety, depression	NA	Confirmatory factor analysis to test the construct validity of the Dari ITQ; multivariate regression predicting PTSD and DSO simultaneously for discriminant validity	PTSD was predicted by pre-migration traumas, post-migration stressors, anxiety, psychological distress; DSO was predicted by post-migration stressors, depression, psychological distress; Asylum seekers showed higher ICD-11 CPTSD symptoms and higher probable diagnostic rates for ICD-11 PTSD, DSM-IV PTSD, anxiety, depression, and psychological distress compared to refugees.	Supports construct validity and cultural sensitivity of Dari ITQ; CPTSD more prevalent than PTSD; differential predictors confirm ICD-11 PTSD/CPTSD distinction: pre-migration trauma and anxiety drive PTSD, while post-migration stressors and depression drive DSO; asylum seekers showed higher symptom burden than refugees across all measures
Bachem et al. (2024)	Examine whether children of asylum-seeking mothers with ICD-11 CPTSD vs PTSD vs no stress disorder display different levels of internalising and externalising problems and explore the role of comorbid depression	Cross-sectional	Eritrean (95.3% born in Eritrea; 4.7% Tigrinya-speaking from other countries)	Israel	Asylum-seeking Eritrean mothers of preschool-aged children	127	23.6%	28.47 ± 3.95	F = 127 (100%)	∼6–12 years post-migration	ITQ (Tigrinyan version)	Maternal trauma exposure; Maternal CPTSD, PTSD, depression	Children's adverse life experiences	Pearson correlations; MANCOVA comparing 3 maternal groups on child internalising/ externalising; MANOVA comparing 4 groups (CPTSD, PTSD, depression only, healthy); post-hoc t-test for CPTSD + depression vs PTSD + depression	CPTSD mothers’ children had higher internalising than PTSD and no-diagnosis children; and higher externalising vs no-diagnosis; 4-group MANOVA significant: CPTSD vs PTSD internalising; CPTSD vs healthy; externalising: CPTSD vs healthy	Maternal CPTSD, not PTSD, drives intergenerational transmission of trauma to child internalising and externalising difficulties; effects observed above and beyond children's own adverse experiences; near-universal CPTSD-depression comorbidity (94.5%) suggests these may be functionally intertwined; DSO symptoms are proposed mechanisms impacting parenting quality and child outcomes
Barbieri et al. (2019)	Compare DSM-5 and ICD-11 PTSD diagnoses and evaluate the discriminant validity of ICD-11 PTSD vs CPTSD in treatment-seeking refugees exposed to complex trauma	Cross-sectional	19 African countries (90.8% West Africa)	Italy	Treatment-seeking refugees (6%) and asylum seekers (94%) exposed to repeated interpersonal trauma	113 Asylum Seekers; 7 Refugees	30%	25.1 ± 6.7	M = 103 (86%); F = 17 (14%)	11.1 **±** 8.9 months	PCL-5 items mapped to ITQ	Demographic characteristics, trauma exposure count, employment status, education level, time spent in Italy	Gender, age, education, trauma exposure, employment status, legal status, months spent in Italy	Diagnostic comparison (DSM-5 vs ICD-11), symptom sensitivity/specificity analyses, LCA, logistic regression (class predictors), linear regression (symptom severity predictors)	LCA identified two distinct latent classes consistent with ICD-11 PTSD (41.7%) and CPTSD (58.3%); no sociodemographic or trauma variable predicted class membership; months in Italy predicted lower PCL-5 scores but not diagnostic class.	Supports discriminant validity of ICD-11 PTSD and CPTSD in asylum seekers/refugees exposed to complex trauma. CPTSD items showed strong sensitivity but low specificity. Complex trauma led to CPTSD in one-third of cases.
Barbieri et al. (2023)	Investigate ICD-11 PTSD and CPTSD symptom profiles and their pre-migration, post-migration, and demographic predictors in asylum-seekers	Cross-sectional	Sudan (85.7%), Cameroon (5.5%), Central African Republic (4.0%), Mali (1.6%), Togo (1.6%), Burkina Faso (0.8%), Palestine (0.8%)	Niger	Treatment-seeking asylum seekers	126	74.6%	26.12 ± 6.88	M = 95 (75.4%) F = 31 (24.6%)	21.66 months (*SD* = 9.74)	ITQ (Arabic, English, French versions)	Age, gender, age at first trauma, functional impairment, traumatic exposures, employment, reception conditions (large vs small centre), family presence	NA	ICD-11 diagnostic prevalence with chi-square gender comparisons; LCA on 12 dichotomous ITQ indicators; multinomial logistic regression with risk factors as predictors	Two-class LCA best fit: CPTSD class 69.0% (high all symptoms), PTSD class 31.0% (high PTSD, moderate DSO except elevated affective dysregulation); 3 predictors of CPTSD vs PTSD class: reception condition - humanitarian site; age of first trauma, younger onset increased CPTSD likelihood; functional impairment	Supports ICD-11 PTSD/CPTSD distinction; both premigration and postmigration factors associated with CPTSD; living in large camps (>1,000) vs small urban facilities (<100) increased CPTSD risk; strong policy implication favouring small-scale, integrated reception.
Benoni et al. (2025)	Describe the socio-ecological context and mental health (psychological distress, PTSD, CPTSD) status of refugees and explore their associations	Cross-sectional	Ukraine	Italy	War refugees	221	13.3%	Median = 35.5, (IQR = 24.7–44.0)	M = 43 (19.2%); F = 181 (80.8%)	Median = 8.7 months (IQR = 6.7–11.6)	ITQ (Ukrainian and Russian versions)	Ecomap stressful edge clusters (None, Micro, Macro/SN, Macro/PS, Exo/Macro), sex, age, chronic diseases	Sex, age chronic disease	Cluster analysis to identify groups of socio-relational con texts; 3 binomial logistic regressions for GHQ-12, PTSD, and CPTSD outcomes with ecomap clusters as predictors	CPTSD odds were 26.3% higher in the Exo/Macro cluster vs none; male sex was protective; PTSD was additionally associated with Macro/SN and Exo/Macro clusters	CPTSD was associated with stressful events in school/ work and public services; Stressful interactions with public services (legal, health) and occupational stressors in the resettlement country were key correlates of trauma-related distress.
Frost et al. (2018)	Evaluate the latent dimensional and categorical structure of ICD-11 PTSD and CPTSD and examine whether trauma history predicts class membership in refugees	Cross-sectional	38 + countries; (44.6% South American, 23% European, 22.6% Asian, 4.6% African, 4.3% North American, 1% other)	United States	Self-identified refugees from US general population survey	308	4.9%	50.94 ± 17.07	M = 158 (51.3%); F = 150 (48.7%)	46.8% reported refugee status > 2 years	AUDADIS-IV items mapped to ICD-11 PTSD/CPTSD criteria	Serious neglect, physical assault, sexual assault, unarmed civilian in conflict, serious accident, natural disaster, cumulative trauma exposure, sex	Age, origin, education, income, duration of refugee status, age of refugee status acquisition	CFA testing 7 competing dimensional models; factor mixture modelling (FMM) combining CFA with LCA (2–6 class solutions); multinomial logistic regression predicting class membership by trauma type and cumulative exposure	Best fitting FMM: correlated 6-factor model with 5 latent classes; two classes consistent with ICD-11 PTSD (15.1%) and CPTSD (13.3%); CPTSD class predicted by interpersonal traumas (neglect, physical assault, sexual assault) and female sex; PTSD class predicted by situational traumas; Dose-response effect between cumulative trauma and CPTSD.	Supports discriminant validity of ICD-11 PTSD and CPTSD at both dimensional and categorical levels; interpersonal and cumulative trauma were key predictors of CPTSD; female refugees substantially more likely to experience CPTSD
Hecker et al. (2018)	Investigate the association between ICD-11 PTSD and DSO symptoms and traumatic experiences, postmigration living difficulties, and social support in a culturally diverse sample of refugees	Cross-sectional	Syria (29.8%), Afghanistan (18.1%), Eritrea (13.8%), Turkey (6.4%), Iraq (5.3%), North Africa (5.3%), Sub-Saharan Africa (4.3%), Other (17%)	Switzerland	Asylum seekers (64.9%); refugees (25.5%); provisional permit (5.3%); no official status (2.1%)	94	21.30%	31.6 ± 10.14	M = 80 (85.1%); F = 14 (14.9%)	NA	ITQ	Trauma exposure, perceived social support, postmigration living difficulties	Gender	2 hierarchical multiple regression analyses: (1) predicting PTSD symptom severity and (2) predicting DSO symptom severity from risk factors	Trauma exposure predicted PTSD symptom severity but not DSO symptom severity; postmigration living difficulties and lack of social support predicted DSO symptom severity but not PTSD symptom severity; gender was not associated with DSO; PTSD model explained 11.2% of variance; DSO model explained 28.5%	PTSD and DSO symptoms show differential associations with risk factors: PTSD is specifically linked to trauma exposure, while DSO is linked to postmigration stressors and social support; Supports the clinical utility of distinguishing PTSD from CPTSD in refugee populations.
Hyland et al. (2018)^a^	Evaluated the discriminant validity of ICD-11 PTSD and CPTSD among treatment-seeking Syrian refugees residing in Lebanon	Cross-sectional	Syria	Lebanon	Treatment-seeking refugees exposed to war-related trauma	110	36.1%	33.02 ± 8.94	M = 22 (19.8%); F = 88 (80.2%)	37.45 ± 14.62 months	ITQ (Arabic version)	Functional impairment, age, years of education, months in Lebanon, gender, marital status, employment, living status, trauma type	NA	Prevalence estimation with chi-square gender comparisons; LCA on PTSD and DSO cluster endorsement; multinomial logistic regression predicting class membership from 9 demographic/ trauma variables	LCA identified three classes: CPTSD (64.5%), PTSD (21.8%), low symptom (13.6%); no gender differences; only functional impairment significantly predicted class membership; Model explained 46.8% of variance	Evidence supporting discriminant validity of the ICD-11 PTSD and CPTSD in Arabic-speaking refugees in a humanitarian setting; prevalence comparable to culturally distinct clinical samples, supporting cross-cultural validity
Ipekci et al. (2023)	Examine the contribution of pre-migration stressors, post-migration stressors, and discrimination to PTSD and CPTSD symptom severity in refugees	Cross-sectional	Iraq (54.2%); Syria (45.7%)	United States	Muslim refugees	94	10.6%	38.5 ± 12.8	NA	4.5 ± 2.3 years	ITQ (Arabic version)	Pre-migration stress exposure, post-migration stress exposure, discrimination	NA	(1) Hierarchical multiple regression predicting PTSD and CPTSD from risk factors; (2) Moderation analysis testing post-migration stressors as moderator of pre-migration → PTSD/CPTSD relationship	Pre- and post-migration stressors together predicted PTSD; adding discrimination was NS; for CPTSD, post-migration stressors and discrimination were significant; post-migration stressors moderated the pre-migration → PTSD relationship: at high post-migration stress, pre-migration stress had less impact; NS for CPTSD	Pre-to-post-migration adversity predicts PTSD, with post-migration context moderating the impact; CPTSD constructs the psychological impact of discrimination; findings highlight importance of post-migration context for both recovery and further traumatisation
Jowett et al. (2021)	Examine rates of ICD-11 PTSD and CPTSD at a specialist trauma service for asylum seekers to assess clinical needs and suitability of existing psychological interventions	Cross-sectional	37 different nations	United Kingdom	Asylum seekers and victims of trafficking referred to specialist trauma service	101	66.23%	34.56 ± 11.01	M = 61 (51.5%); F = 49 (48.50%)	NA	ITQ	Trauma type (torture, sexual abuse/rape, exploitation, domestic violence, other extreme violence), trafficking status, age, gender, post-migration living difficulties (basic survival, healthcare, relationships, integration, housing), clinical distress	NA	ANOVA comparing three ITQ diagnostic groups (CPTSD, PTSD, no diagnosis) on age, clinical distress, ITQ subscales, post-migration living difficulties subscales; Chi square comparing diagnostic groups on gender, trafficking status, and trauma type	CPTSD group had higher clinical distress than no-diagnosis; CPTSD had higher negative self-concept, disturbed relationships and post-migration living difficulties than PTSD; both PTSD and CPTSD had higher healthcare needs than no-diagnosis.	High prevalence of CPTSD in multiply traumatised asylum-seekers; CPTSD associated with greater overall clinical distress and post-migration relationship difficulties compared to PTSD, highlights need for multicomponent treatment addressing both PTSD and DSO symptoms, including culturally sensitive approaches and flexible treatment pathways.
Karstoft et al. (2024)^b^	Estimate prevalence of ICD-11 PTSD and CPTSD among displaced persons and examine associations with war-related and other traumatic exposures	Cross-sectional	Ukraine	Denmark	Displaced refugees	6,993	13.5%	Median = 38 (IQR = 18)	M = 1,589 (22.7%); F = 5,404 (77.3%)	∼10–13 months for majority	ITQ (Ukrainian and Russian versions)	War exposures (combat exposure, hometown affected, presence during war, prior war exposure, loss of relatives), interpersonal and non-interpersonal traumatic events, education, employment/study, family location	Age, gender	Binomial logistic regression for combined (C)PTSD vs. no diagnosis; multinomial logistic regression for PTSD vs. CPTSD vs. no diagnosis; Stepwise: univariate screening (*p* < .10), then multivariable	Associated with both, PTSD and CPTSD: education, loss of family/friends, interpersonal and non-interpersonal potentially traumatic events; PTSD only: actions of war near hometown, home damaged, present during war, direct combat exposure; CPTSD only: younger age, not employed/ studying, lived in war-affected area before 2022; gender and interpersonal potentially traumatic events effects stronger for CPTSD than PTSD	PTSD and CPTSD share common predictors but show distinct patterns: direct/acute war exposure predicts PTSD only, supporting a dose-response model for PTSD. Protracted war exposure and unemployment predict CPTSD only, consistent with theory that CPTSD arises from prolonged trauma. Interpersonal traumatic events more strongly associated with CPTSD, supporting ICD-11 conceptualisation
Karstoft et al. (2025)^b^	Test prospective associations between pre-migration war exposure, post-migration living difficulties and social support with ICD-11 PTSD/CPTSD	Prospective cohort (two-wave design)	Ukraine	Denmark	Refugees settled during first year after invasion	4,229	12.2%	Median = 40 (IQR = 22)	M = 995 (23.5%); F = 3,234 (76.5%)	∼12-22 months post-displacement	ITQ (Ukrainian and Russian versions)	War exposures, post-migration living difficulties (health, refugee process, family concerns, general), perceived social support	Age, gender, education, employment status, additional pre-migration trauma exposures	Multinomial logistic regression with risk factors as predictors and social support interaction (moderation)	Family, health, and general post-migration living difficulties and social support associated with CPTSD; health, family and war exposures with PTSD; Social support moderated refugee and family post-migration living difficulties → PTSD, but noy for CPTSD; female gender associated with both, PTSD and CPTSD	Differential risk factor patterns for PTSD and CPTSD, suggesting different underlying mechanisms. Post-migration living difficulties are important for both, but more strongly associated with CPTSD; studying PTSD and CPTSD as separate outcomes in refugee populations yields distinct and clinically relevant insights
Liddell et al. (2019)	Examine PTSD/CPTSD symptom structure and demographic, premigration, and postmigration predictors in refugees	Cross-sectional	25 countries; primarily Iran (34.8%), Iraq (20.5%), Afghanistan (6.3%), Sri Lanka (6.3%); also, Syria, Vietnam, Bangladesh, Ethiopia, and others	Australia	Mixed treatment-seeking and community-based refugees	112	29.5%	37.7 ± 11.5	M = 74 (66.1%); F = 38 (33.9%)	NA	PSS-I for 6 ICD-11-aligned PTSD symptoms; precursor version of ITQ for DSO	Sex, age Cumulative trauma load, visa status, exposure to torture or sexual violence	NA	LCA on 11 dichotomised symptom indicators (6 PTSD, 5 DSO) to identify symptom profiles; multinomial logistic regression on derived classes with risk factors as predictors.	4-class model: (1) CPTSD (29.5%): high PTSD + high DSO; (2) PTSD-only (23.5%): high PTSD, low-moderate DSO; (3) affective dysregulation (AD) class (31.9%): high AD, low PTSD, moderate DSO; (4) Low symptom (15.1%); cumulative trauma predicted CPTSD and PTSD; female sex predicted all classes; insecure visa predicted CPTSD and AD but not PTSD; CPTSD vs PTSD: insecure visa predicted CPTSD over PTSD; older age predicted PTSD over CPTSD	LCA supports ICD-11 distinction between PTSD and CPTSD in a diverse refugee sample, a novel fourth AD class (31.9%) was identified, characterised by high AD without PTSD symptoms; post-migration contextual stressors specifically contribute to DSO symptomatology; findings support both pre-migration trauma and post-migration contextual factors as correlates of CPTSD symptom profiles in refugees
McGinty et al. (2023)	Determine prevalence of ICD-11 PTSD and CPTSD among Ukrainian IDPs, and examine how coping styles relate to PTSD and CPTSD	Cross-sectional	Ukraine	Ukraine	IDPs	2,198	7.8%	45 ± 16.99	M = 702 (31.9%); F = 1,496 (68.1%)	17.49 ± 4.49 months	ITQ	Coping styles (problem-focused, emotion-focused, avoidant, stoic), sex, diagnostic group	NA	Exploratory factor analysis to derive coping-style factors for comparison across ICD-11 PTSD/CPTSD diagnostic groups; ANOVA (diagnostic status × sex) for coping style differences	Avoidant coping was higher in PTSD and CPTSD than those with no diagnosis; significant sex × diagnosis interaction: for males, avoidant coping was elevated equally in PTSD and CPTSD; for females, avoidant coping increased stepwise (no diagnosis → PTSD → CPTSD)	PTSD (13.1%) was more prevalent than CPTSD (7.8%) among Ukrainian IDPs. Interventions targeting avoidant coping may reduce traumatic stress burden in war-affected IDPs. Avoidant coping may help differentiate CPTSD from PTSD specifically in women
Rawers et al. (2024)	Identify trauma exposure classes and examine associations with PTSD/CPTSD and other psychopathology in refugees	Cross-sectional	Syria	Turkey	Community-based refugees	593	11.5%	33.89 ± 11.45	M = 309 (52.1%); F = 284 (47.9%)	NA	ITQ (Arabic version)	Age, gender, education, marital status, income	NA	LCA; multinomial logistic regression for demographic predictors of class membership; associations between class membership and probable ICD-11 PTSD/CPTSD and DSM-IV MDD/GAD diagnoses	Three-class solution: ‘multiple traumas’, ‘war and human suffering’, ‘low exposure, combat-exposed’; CPTSD rates by class: multiple traumas 18.28%, war and human suffering 9.47%, low exposure 10.89%. No differences in probability of CPTSD, PTSD, or MDD between classes; ‘multiple traumas’ class had a higher probability of GAD compared to ‘low exposure’ class	Trauma exposure class membership did not predict CPTSD, PTSD, or MDD; post-migration factors may be more strongly associated with mental health outcomes than pre-migration trauma exposure patterns; CPTSD prevalence was nearly double that of PTSD; Highlights the need to incorporate post-migration variables in research
Silove et al. (2017)^c^	Examine structural relationship between ICD-11 PTSD and CPTSD with complex grief and MDD in a persecuted refugee population	Cross-sectional	West Papua	Papua New Guinea	Community-based refugees	230	3%	37.0 ± 9.80	M = 137 (59.5%); F = 93 (40.4%)	Among those born in West Papua (46.5%, n = 107): mean 27 years	R-MHAP	NA	NA	Pearson correlations between symptom counts of CPTSD, PTSD, MDD, and complex grief; Confirmatory factor analysis comparing: (1) one-factor model (combined PTSD, CPTSD), (2) two-factor model (separate PTSD, CPTSD)	Symptom count correlations: PTSD–CPTSD r = 0.78; CPTSD–MDD r = 0.42; CPTSD–CG r = 0.19. PTSD and CPTSD symptoms loaded onto a single traumatic stress factor	Single traumatic stress factor combining PTSD and CPTSD symptom domains, suggests that the theoretical distinction between PTSD and CPTSD may not hold in populations exposed to multiple traumas associated with mass conflict and human rights violations.
Tay et al., (2015)^c^	Examine factorial structure of PTSD (DSM-IV, DSM-5, ICD-10, ICD-11) and CPTSD (proposed ICD-11) in a transcultural refugee population	Cross-sectional	West Papua	Papua New Guinea	Community-based refugees	230	3%	37 ± 9.80	M = 137 (59.5%); F = 93 (40.4%)	Among those born in West Papua (46.5%): 27 ± 10.28 years	R-MHAP mapped to DSM-IV/5 and ICD-10/11 criteria	5 trauma exposures (conflict-related, witnessing murders, traumatic losses, access to emergency care, childhood adversities); ADAPT pillars (safety /security, justice/ injustice)	Age, marital status	Confirmatory factor analysis testing DSM-IV 3-, DSM-5 4/5/6/7-, ICD-10 3-, ICD-11 3-factor PTSD models, and ICD-11 6-factor C-PTSD model; MIMIC examined risk factors as predictors of PTSD/C-PTSD symptom domains with interaction effects	ICD-10/−11 3-factor PTSD models yielded good fit; DSM-IV/−5 showed poor fit; CPTSD: six individual factors identified (good first-order fit, but second-order model showed poor fit); no unitary C-PTSD construct supported; witnessing murder × injustice associated with intrusion and avoidance domains of PTSD and CPTSD but not with the three unique DSO domains	Supports ICD PTSD construct cross-culturally; CPTSD did not form a unitary construct; individual DSO domains identifiable but did not cohere under a single higher-order factor; findings question cross-cultural applicability of CPTSD as a distinct diagnosis
Tay et al. (2018)	Test theoretically supported factorial models of ICD-11 CPTSD and examine concurrent validity with trauma exposure, post-migration stressors, and functional impairment	Cross-sectional	West Papua	Papua New Guinea	Community-based refugees	486	9.3%	35.8 ± 14.3	M = 272 (56%); F = 214 (44%)	Among those born in West Papua (56.8%): 15.6 ± 10.7 years	R-MHAP mapped to ICD-11 CPTSD	Trauma exposure, post-migration living difficulties, functional impairment	NA	Confirmatory factor analysis comparing 4 CPTSD and 3 PTSD models; Multiple linear regression for concurrent validity of CPTSD symptom score	Six-factor CPTSD model yielded best fit; all PTSD-only models showed poor fit; CPTSD score associated with trauma exposure, post-migration living difficulties, functional impairment	Supports cross-cultural validity of the ICD-11 six-factor CPTSD structure in a refugee population with no prior exposure to western trauma concepts; CPTSD may be particularly relevant for refugees exposed to prolonged persecution
Vallières et al. (2018)^a^	Evaluate ICD-11 PTSD and CPTSD prevalence, factor structure and clinical utility of the ITQ in refugees	Cross-sectional	Syria	Lebanon	Treatment-seeking refugees	112	36.1%	33.02 ± 8.94	M = 22 (19.8%); F = 90 (80.20%)	37.45 ± 14.62 months	ITQ (Arabic version)	Sex, age	NA	Confirmatory factor analysis to assess the factor structure of PTSD/CPTSD symptoms; prevalence estimation with chi-square and t-tests for sex/age differences; thematic analysis	Best-fitting model: two-factor (PTSD + DSO) with affective dysregulation split into hyper/ hypoactivation; CPTSD (36.1%) more common than PTSD (25.2%); no sex or age differences; psychotherapists reported ITQ was clinically useful but noted literacy and cultural adaptation challenges	Supporting ICD-11 PTSD/CPTSD factor structure among Middle Eastern refugees and ITQ clinical utility in humanitarian settings; prevalence comparable to other treatment-seeking refugee samples, supporting cross-cultural applicability
Vang et al. (2019)	Test ICD-11 PTSD/CPTSD validity in culturally heterogeneous treatment-seeking refugees	Cross-sectional	24 countries; most represented: Syria (42.9%), Bosnia (17.6%), Afghanistan (8.8%), Kosovo (7.4%)	Denmark	Treatment-seeking refugees	284	66.9%	40.94 ± 9.77	M = 149 (52.5%); F = 135 (47.5%)	34.8% 1–5 years, 12.8% 5–10 years, 17.7% 10–20 years, 31.2% > 20 years	ITQ	NA	Age, gender, citizenship status, index trauma type, marital status, occupational status, need for interpreter, time since trauma, region of origin	LCA to test the validity of the ICD-11 PTSD/ CPTSD distinction; Chi-square cross-tabulation of LCA classes with probable ICD-11 diagnosis; multinomial logistic regression for covariate–class associations	Two-class model best fit; class 1 (CPTSD, 87%): high endorsement across all clusters; class 2 (PTSD, 13%): elevated PTSD symptoms and affective dysregulation, lower negative self-concept and disturbed relationships; Probable diagnosis: 66.9% CPTSD, 25% PTSD, 8.1% no diagnosis; No covariate predictors of class membership	Supports ICD-11 PTSD/ CPTSD distinction in a culturally heterogeneous treatment-seeking refugee sample; CPTSD was the predominant presentation, suggesting need for treatment beyond standard PTSD-focused interventions. affective dysregulation elevated even in PTSD class

Notes. ADAPT: Adaptation and Development After Persecution and Trauma; AD: Affective Dysregulation; ANOVA: Analysis of Variance; AUDADIS-IV: Alcohol Use Disorder and Associated Disabilities Interview Schedule-IV; CFA: Confirmatory Factor Analysis; CI: Confidence Interval; CPTSD: Complex Post-Traumatic Stress Disorder; DSM-IV: Diagnostic and Statistical Manual of Mental Disorders, 4th Edition; DSM-5: Diagnostic and Statistical Manual of Mental Disorders, 5th Edition; DSO: Disturbances in Self-Organisation; EFA: Exploratory Factor Analysis; F: Female; FMM: Factor Mixture Modelling; GAD: Generalised Anxiety Disorder; GHQ-12: General Health Questionnaire-12; ICD-10: International Classification of Diseases, 10th Revision; ICD-11: International Classification of Diseases, 11th Revision; IDPs: Internally Displaced Persons; IQR: Interquartile Range; ITQ: International Trauma Questionnaire; LCA: Latent Class Analysis; M: Male; MANCOVA: Multivariate Analysis of Covariance; MANOVA: Multivariate Analysis of Variance; MDD: Major Depressive Disorder; MIMIC: Multiple Indicators Multiple Causes model; NA: Not Available; NS: Non-significant; OR: Odds Ratio; PCL-5: PTSD Checklist for DSM-5; PSS-I: PTSD Symptom Scale–Interview; PTSD: Post-Traumatic Stress Disorder; R-MHAP: Refugee Mental Health Assessment Package; SD: Standard Deviation. Superscript letters denote overlapping samples: studies sharing the same letter (ᵃ, ᵇ, ᶜ) drew from the same or substantially overlapping participant pools and will therefore contribute only one prevalence estimate each to the meta-analysis to avoid non-independence of effect sizes.

Total sample sizes across the 20 studies ranged from 94 (Hecker et al., 2018; Ipekci, 2023) to 6,993 (Karstoft et al., 2024), with a combined total of 12,504 participants across all independent samples. The majority of studies sampled refugees (*k* = 13) (Benoni et al., 2025; Frost et al., 2019; Hyland et al., 2018; Ipekci, 2023; Karstoft et al., 2024; 2025; Liddell et al., 2019; Rawers et al., 2024; Silove et al., 2017; Tay et al., 2015; 2018; Vallières et al., 2018; Vang et al., 2020), with fewer studies focusing on asylum seekers (*k* = 3) (Bachem et al., 2024; Barbieri et al., 2023; Jowett et al., 2021), IDPs (*k* = 1) (McGinty et al., 2023), or mixed samples of refugees and asylum seekers (*k* = 3) (Andisha et al., 2023; Barbieri et al., 2019; Hecker et al., 2018). Seven studies recruited treatment-seeking participants from clinical or specialist trauma services (Barbieri et al., 2019; 2023; Hyland et al., 2018; Vallières et al., 2018; Vang et al., 2020) or a mixture of treatment-seeking and community-based participants (Andisha et al., 2023; Liddell et al., 2019), while the remaining 13 recruited community-based or population-based samples (Bachem et al., 2024; Benoni et al., 2025; Frost et al., 2019; Hecker et al., 2018; Ipekci, 2023; Jowett et al., 2021; Karstoft et al., 2024; 2025; McGinty et al., 2023; Rawers et al., 2024; Silove et al., 2017; Tay et al., 2015; 2018).

#### 3.2.2. Participant demographics

Where reported as a mean, participant average age ranged from 25.1 years (*SD* = 6.7) (Barbieri et al., 2019) to 50.9 years (*SD* = 17.1) (Frost et al., 2019). Three studies reported age as a median instead, ranging from 35.5 years (IQR = 24.7–44.0) (Benoni et al., 2025) to 40 years (IQR = 22) (Karstoft et al., 2025). The proportion of male participants varied considerably across studies, from 0% (Bachem et al., 2024), comprising exclusively female asylum-seeking mothers, to 86% (Barbieri et al., 2023). The majority of studies (*k* = 12) included both male and female participants, with most samples having a higher proportion of males (Andisha et al., 2023; Barbieri et al., 2019; 2023; Frost et al., 2019; Hecker et al., 2018; Jowett et al., 2021; Liddell et al., 2019; Rawers et al., 2024; Silove et al., 2017; Tay et al., 2015; 2018; Vang et al., 2020). Notable exceptions included studies of Ukrainian displaced populations, which consistently reported a higher proportion of females (68–81%) (Benoni et al., 2025; Karstoft et al., 2024; Karstoft et al., 2025; McGinty et al., 2023), while one study did not report sex distribution (Ipekci, 2023).

#### 3.2.3. Geographic distribution

Participants originated from a wide range of displacement contexts. The most frequently represented regions of origin were Ukraine (*k* = 4) (Benoni et al., 2025; Karstoft et al., 2024; 2025; McGinty et al., 2023), Syria (*k* = 4) (Hyland et al., 2018; Ipekci, 2023; Vallières et al., 2018; Vang et al., 2020), West Papua (*k* = 3) (Silove et al., 2017; Tay et al., 2015; Tay et al., 2018), and sub-Saharan Africa (*k* = 3) (Bachem et al., 2024; Barbieri et al., 2019; 2023). Several studies drew from mixed-origin samples (Frost et al., 2019; Hecker et al., 2018; Jowett et al., 2021; Liddell et al., 2019; Vang et al., 2020). Resettlement countries included Denmark (*k* = 3) (Karstoft et al., 2024; 2025; Vang et al., 2020), Papua New Guinea (*k* = 3) (Silove et al., 2017; Tay et al., 2015; 2018), Italy (*k* = 2) (Barbieri et al., 2019; Benoni et al., 2025), Lebanon (*k* = 2) (Hyland et al., 2018; Vallières et al., 2018), the US (*k* = 2) (Frost et al., 2019; Ipekci, 2023), and one study each in Australia (Liddell et al., 2019), Austria (Andisha et al., 2023), Israel (Bachem et al., 2024), Niger (Barbieri et al., 2023), Switzerland (Hecker et al., 2018), Turkey (Rawers et al., 2024), and the UK (Jowett et al., 2021). One study examined internally displaced persons within Ukraine (McGinty et al., 2023). Examining the country level income is one of the environmental factors that determine the psychological reactions of the participants.

#### 3.2.4. Time since displacement

Time since displacement was reported in 12 of the 20 studies, and varied substantially across samples. Durations ranged from approximately 10–22 months in recently displaced Ukrainian populations (Benoni et al., 2025; Karstoft et al., 2024; 2025; McGinty et al., 2023) to over 27 years among West Papuan refugees in protracted displacement (Silove et al., 2017; Tay et al., 2015).

#### 3.2.5. CPTSD measurements

A critical distinction emerged in how CPTSD was operationalised across studies. The majority of studies (*k* = 14) used the ITQ or a validated language adaptation thereof (Arabic, Dari, Tigrinya, Ukrainian, or Russian versions) (Andisha et al., 2023; Bachem et al., 2024; Barbieri et al., 2023; Benoni et al., 2025; Hecker et al., 2018; Hyland et al., 2018; Ipekci, 2023; Jowett et al., 2021; Karstoft et al., 2024; 2025; McGinty et al., 2023; Rawers et al., 2024; Vallières et al., 2018; Vang et al., 2020). The remaining six studies instead mapped items from other instruments onto ICD-11 CPTSD criteria: (Barbieri et al., 2019) mapped items from the PTSD Checklist for DSM-5 (PCL-5), (Frost et al., 2019) used the Alcohol Use Disorder and Associated Disabilities Interview Schedule-IV (AUDADIS-IV), (Liddell et al., 2019) combined the PTSD Symptom Scale -Interview (PSS-I) with a precursor version of the ITQ for DSO symptoms, and (Silove et al., 2017; Tay et al., 2016; Tay et al., 2018) used the Refugee Mental Health Assessment Package (R-MHAP).

#### 3.2.6. CPTSD prevalence

Reported CPTSD prevalence estimates varied widely across studies, ranging from 3.0% (Silove et al., 2017; Tay et al., 2015) to 74.6% (Barbieri et al., 2023). This variability likely reflected differences in sample characteristics, recruitment context, and measurement approach. Among treatment-seeking samples, CPTSD prevalence was generally higher, ranging from 30.0% (Barbieri et al., 2019) to 74.6% (Barbieri et al., 2023). The highest rates (74.6%) were observed among treatment-seeking asylum seekers in Niger (Barbieri et al., 2023), 66.9% among treatment-seeking refugees in Denmark (Vang et al., 2020), and 66.2% among asylum seekers and survivors of trafficking referred to a specialist trauma service in the UK (Jowett et al., 2021). Among community-based samples, prevalence was generally lower, ranging from 3% (Silove et al., 2017; Tay et al., 2015) to 23.6% (Bachem et al., 2024), with several studies reporting estimates between 7.8% and 13.5% (Benoni et al., 2025; Frost et al., 2019; Ipekci, 2023; Karstoft et al., 2024; 2025; Rawers et al., 2024; Tay et al., 2018). Two studies recruited mixed treatment-seeking and community-based samples with prevalence rates of 20.7% (Andisha et al., 2023) and 29.5% (Liddell et al., 2019). Prevalence also appeared to differ by measurement approach. Among the 14 studies using the ITQ directly, estimates ranged from 7.8% (McGinty et al., 2023) to 74.6% (Barbieri et al., 2023). Among the six studies that mapped other instruments onto ICD-11 criteria, estimates ranged from 3.0% (Silove et al., 2017; Tay et al., 2015) to 30.0% (Barbieri et al., 2019).

#### 3.2.7. Risk factors

Risk factors examined across studies varied considerably in type and frequency. The most commonly examined variables included sex (*k* = 15) (Andisha et al., 2023; Barbieri et al., 2019; 2023; Benoni et al., 2025; Frost et al., 2019; Hecker et al., 2018; Hyland et al., 2018; Jowett et al., 2021; Karstoft et al., 2024; 2025; Liddell et al., 2019; McGinty et al., 2023; Rawers et al., 2024; Vallières et al., 2018; Vang et al., 2020), age (*k* = 14) (Andisha et al., 2023; Barbieri et al., 2019; 2023; Benoni et al., 2025; Frost et al., 2019; Hyland et al., 2018; Jowett et al., 2021; Karstoft et al., 2024; 2025; Liddell et al., 2019; Rawers et al., 2024; Tay et al., 2015; Vallières et al., 2018; Vang et al., 2020), trauma exposure or cumulative trauma load (*k* = 10) (Barbieri et al., 2019; 2023; Frost et al., 2019; Hecker et al., 2018; Jowett et al., 2021; Karstoft et al., 2024; 2025; Liddell et al., 2019; Tay et al., 2015; 2018), and post-migration living difficulties (*k* = 4) (Hecker et al., 2018; Jowett et al., 2021; Karstoft et al., 2025; Tay et al., 2018). Several studies examined pre-migration stressors alongside post-migration factors (Hecker et al., 2018; Ipekci, 2023; Karstoft et al., 2025; Liddell et al., 2019). Other risk factors examined in individual studies included coping styles (McGinty et al., 2023), reception conditions (Barbieri et al., 2023), discrimination (Ipekci, 2023), perceived social support (Hecker et al., 2018; Karstoft et al., 2025), visa or legal status (Liddell et al., 2019), and socio-ecological stressors (Benoni et al., 2025). A recurring finding across multiple studies was that post-migration living difficulties were more strongly associated with DSO symptoms than with core PTSD symptoms, while trauma exposure was more consistently associated with PTSD symptoms (Hecker et al., 2018; Karstoft et al., 2024; 2025; Liddell et al., 2019).

### 3.3. Risk of bias

Pre-consensus inter-rater reliability on the JBI Critical Appraisal Checklist was moderate (*ICC*[A,1] = 0.58, 95% CI [0.21, 0.81]). All disagreements were resolved through consensus discussion to produce final agreed scores. Final JBI quality scores ranged from 4 to 9 (out of a possible 9). Eight studies were rated as low risk of bias (scored 7–9) (Bachem et al., 2024; Benoni et al., 2025; Karstoft et al., 2024; 2025; McGinty et al., 2023; Silove et al., 2017; Tay et al., 2015; 2018), and 12 as moderate risk of bias (scores 4–6) (Andisha et al., 2023; Barbieri et al., 2019; 2023; Frost et al., 2019; Hecker et al., 2018; Hyland et al., 2018; Ipekci, 2023; Jowett et al., 2021; Liddell et al., 2019; Rawers et al., 2024; Vallières et al., 2018; Vang et al., 2020). No studies were rated as high risk of bias. The full quality appraisal ratings for each study are presented in Supplementary Table 2.

### 3.3.1. Instrument validation

Fourteen studies used the ITQ (Andisha et al., 2023; Bachem et al., 2024; Barbieri et al., 2019; 2023; Benoni et al., 2025; Hecker et al., 2018; Hyland et al., 2018; Jowett et al., 2021; Karstoft et al., 2024; 2025; McGinty et al., 2023; Rawers et al., 2024; Vallières et al., 2018; Vang et al., 2020) and six mapped items from other instruments onto the ICD-11 CPTSD criteria (Barbieri et al., 2019; Frost et al., 2019; Liddell et al., 2019; Silove et al., 2017; Tay et al., 2015; 2018) (Supplementary Table 2B). Written instrument versions in 15 distinct languages were used across studies, and nine studies administered instruments in more than one language; three studies additionally relied on interpreters for a substantial proportion of participants (Hecker et al., 2018; Jowett et al., 2021; Vang et al., 2020). Arabic (Barbieri et al., 2019; 2023; Hecker et al., 2018; Hyland et al., 2018; Jowett et al., 2021; Rawers et al., 2024; Vallières et al., 2018; Vang et al., 2020) and English (Barbieri et al., 2019; 2023; Frost et al., 2019; Hecker et al., 2018; Jowett et al., 2021; Liddell et al., 2019; Tay et al., 2018) were the most frequent, followed by Ukrainian (Benoni et al., 2025; Karstoft et al., 2024; 2025; McGinty et al., 2023), French (Barbieri et al., 2019; 2023; Hecker et al., 2018), Russian (Benoni et al., 2025; Karstoft et al., 2024; 2025; McGinty et al., 2023), and Bahasa Indonesian (Silove et al., 2017; Tay et al., 2015; 2018). Bosnian (Vang et al., 2020), Danish (Vang et al., 2020), Dari (Andisha et al., 2023), German (Hecker et al., 2018), Spanish (Hecker et al., 2018), Tamil (Hecker et al., 2018), Tigrinya (Bachem et al., 2024), Tok Pisin (Tay et al., 2018), and Turkish (Hecker et al., 2018) were each used in a single study. Nine studies explicitly reported a translation and/or cultural adaptation process (Andisha et al., 2023; Bachem et al., 2024; Hecker et al., 2018; Hyland et al., 2018; Silove et al., 2017; Tay et al., 2015; 2018; Vallières et al., 2018; Vang et al., 2020), four used original English-language versions requiring no translation (Barbieri et al., 2019; Frost et al., 2019; Jowett et al., 2021; Liddell et al., 2019), and the remaining seven provided no details regarding translation or cultural adaptation (Barbieri et al., 2023; Benoni et al., 2025; Ipekci, 2023; Karstoft et al., 2024; 2025; McGinty et al., 2023; Rawers et al., 2024).

Psychometric evidence for the version administered was reported for the study sample in 15 of the 20 studies and was largely confined to internal consistency (Supplementary Table 2B): reliability was reported separately for the PTSD and DSO clusters in 13 studies [12 independent samples): across the ITQ-based and mapped self-report instruments Cronbach’s α (or composite reliability) ranged from .70 to .89 for PTSD and from .75 to .94 for DSO (Andisha et al., 2023; Bachem et al., 2024; Barbieri et al., 2023; Benoni et al., 2025; Hecker et al., 2018; Hyland et al., 2018; Liddell et al., 2019; Rawers et al., 2024; Vallières et al., 2018; Vang et al., 2020), and reached .88–.97 for the dichotomous, interviewer-administered R-MHAP (Silove et al., 2017; Tay et al., 2015; 2018). Two further studies reported reliability for total scores only (α = .81 for the mapped PCL-5 items [Barbieri et al., 2019]; α = .89 [McGinty et al., 2023]), and five reported no psychometric statistics for their sample (Frost et al., 2019; Ipekci, 2023; Jowett et al., 2021; Karstoft et al., 2024; 2025). Reliability at the level of individual symptom clusters was reported in only one study and was weak for hypervigilance and affective dysregulation (α = .55 and .53; [Vang et al., 2020]). Test–retest stability was examined in a single sample (six-month follow-up, *n* = 101; [Tay et al., 2015]). Confirmatory factor analysis of the CPTSD instrument in the study sample was reported for five independent samples: the ICD-11 two-factor second-order structure of PTSD and DSO was supported for the Dari ITQ (CFI .965, TLI .950, RMSEA .056; [Andisha et al., 2023]) and the Arabic ITQ (Vallières et al., 2018), and a correlated six-factor structure fitted the mapped AUDADIS-IV items (Frost et al., 2019), whereas in the three West Papuan studies using the culturally adapted R-MHAP, a unitary or correlated six-factor solution was preferred, and higher-order PTSD/DSO models fitted poorly or failed to converge (Silove et al., 2017; Tay et al., 2015; Tay et al., 2018). Population-specific validation among forcibly displaced populations was limited. The English, Arabic, Dari, Danish, and Bosnian versions of the ITQ, and the Bahasa Indonesian version of the R-MHAP, had been validated in forcibly displaced populations. Overall, 10 of the 20 studies (8 of the 17 independent samples in the meta-analysis) were rated as having used an instrument validated in the language and population studied.

### 3.4. Meta-analysis: pooled prevalence of CPTSD

Using a random-effects meta-analysis (*k* = 17, *n* = 12,504), the pooled prevalence of CPTSD among refugees, asylum seekers, and IDPs was 20.9% (95% CI [12.6%, 32.6%], see Figure 2). The 95% prediction interval was [1.7%, 80.3%], indicating that the true prevalence in a comparable future study could plausibly fall almost anywhere within this range. Heterogeneity was substantial (*I^2^* = 98.3%, *τ^2^* = 1.57, *Q*(16) = 936.97, *p* < .001). The subgroup analysis by measurement tool did not reveal a significant difference, *Q*(1) = 2.64, *p* = .104. Studies using the ITQ (*k* = 12) yielded a pooled prevalence of 26.3% (95% CI [15.1%, 41.7%], *I^2^* = 98.7%, *τ^2^* = 1.47, *Q*(11) = 843.62, *p* < .001) while studies using mapped instruments (*k* = 5) yielded a pooled prevalence of 11.3% (95% CI [4.3%, 26.4%], *I^2^* = 95.6%, *τ^2^* = 1.34, *Q*(4) = 90.67, *p* < .001; see Supplementary Figure 1). A subgroup analysis based on recruitment context indicated a significant difference in CPTSD prevalence between groups, *Q*(2) = 31.72, *p* < .001. The pooled prevalence in community-based samples (*k* = 10) was 10.6% (95% CI [7.4%, 15.0%], *I^2^* = 91.5%, *τ^2^* = 0.36, *Q*(9) = 105.72, *p* < .001). In treatment-seeking samples (*k* = 5), the pooled prevalence was substantially higher at 55.2% (95% CI [36.7%, 72.3%], *I^2^* = 95.1%, *τ^2^* = 0.7, *Q*(4) = 81.35, *p* < .001). Mixed samples (*k* = 2) yielded an intermediate pooled prevalence of 24.3% (95% CI [16.9%, 33.8%], *I^2^* = 71.8%, *τ^2^* = 0.08, *Q*(1) = 3.55, *p* = .059; see Supplementary Figure 2). Given that this subgroup comprised only two studies, these estimates should be interpreted with caution. The subgroup analysis of CPTSD prevalence based on displacement also revealed significant between-group differences, *Q*(3) = 64.0, *p* < .001. The pooled prevalence among refugees (*k* = 10) was 15.2% (95% CI [7.8%, 27.4%], *I^2^* = 98.2%, *τ^2^* = 1.4, *Q*(9) = 498.86, *p* < .001). Among asylum seekers (*k* = 3), the pooled prevalence was substantially higher at 54.8% (95% CI [23.8%, 82.5%], *I^2^* = 97%, *τ^2^* = 1.4, *Q*(2) = 66.77, *p* < .001). Mixed samples (*k* = 3; including both refugees and asylum seekers) yielded a pooled prevalence of 23.6% (95% CI [18.3%, 29.9%], *I^2^* = 54.4%, *τ^2^* = 0.04, *Q*(2) = 4.38, *p* = .111). For IDPs, only one study was available (*k* = 1), reporting a prevalence of 7.8% (95% CI [6.7%, 9.0%]); therefore, no pooled estimate or heterogeneity statistics could be calculated (see Supplementary Figure 3). A subgroup analysis based on the income classification of the country of resettlement (dichotomised as high-income versus low- and middle-income countries because too few studies were available for finer categories) was not statistically significant, *Q*(1) = 0.37, *p* = .544 (Supplementary Figure 4). The pooled prevalence among high-income countries (k = 11) was 23.6% (95% CI [13.8%, 37.3%], *I^2^* = 98.3%, *τ^2^* = 1.18, *Q*(10) = 571.51, *p* <.001  . Among low-income countries (k = 6) the pooled prevalence was 16.5% (95% CI [5.20%, 41.7%]), *I^2^* = 98.5%, *τ^2^* = 2.53, Q(5) = 333.09, *p* < .001). Subgroup analysis by instrument validation status (Supplementary Figure 5) was also not significant, *Q*(1) = 0.19, *p* = .661. Among studies using an instrument validated in the language and population studied (k = 8), the pooled prevalence was 18.6% (95% CI [8.70%, 35.3%], *I^2^* = 98.1%, *τ^2^* = 1.53, *Q*(7) = 373.66, *p* < .001*)*. Similarly, among studies using an instrument without such validation (*k* = 9) the pooled prevalence was 23.1% (95% CI [11.1%, 42.0%]), *I^2^* = 98.3%, *τ^2^* = 1.77, *Q*(8) = 462.86, *p* < .001. Meta-regression analyses revealed that neither mean age, proportion of female participants, nor time since displacement significantly moderated CPTSD prevalence, though the limited number of studies available for time since displacement (*k* = 8) constrains interpretability.

**Figure 2.**
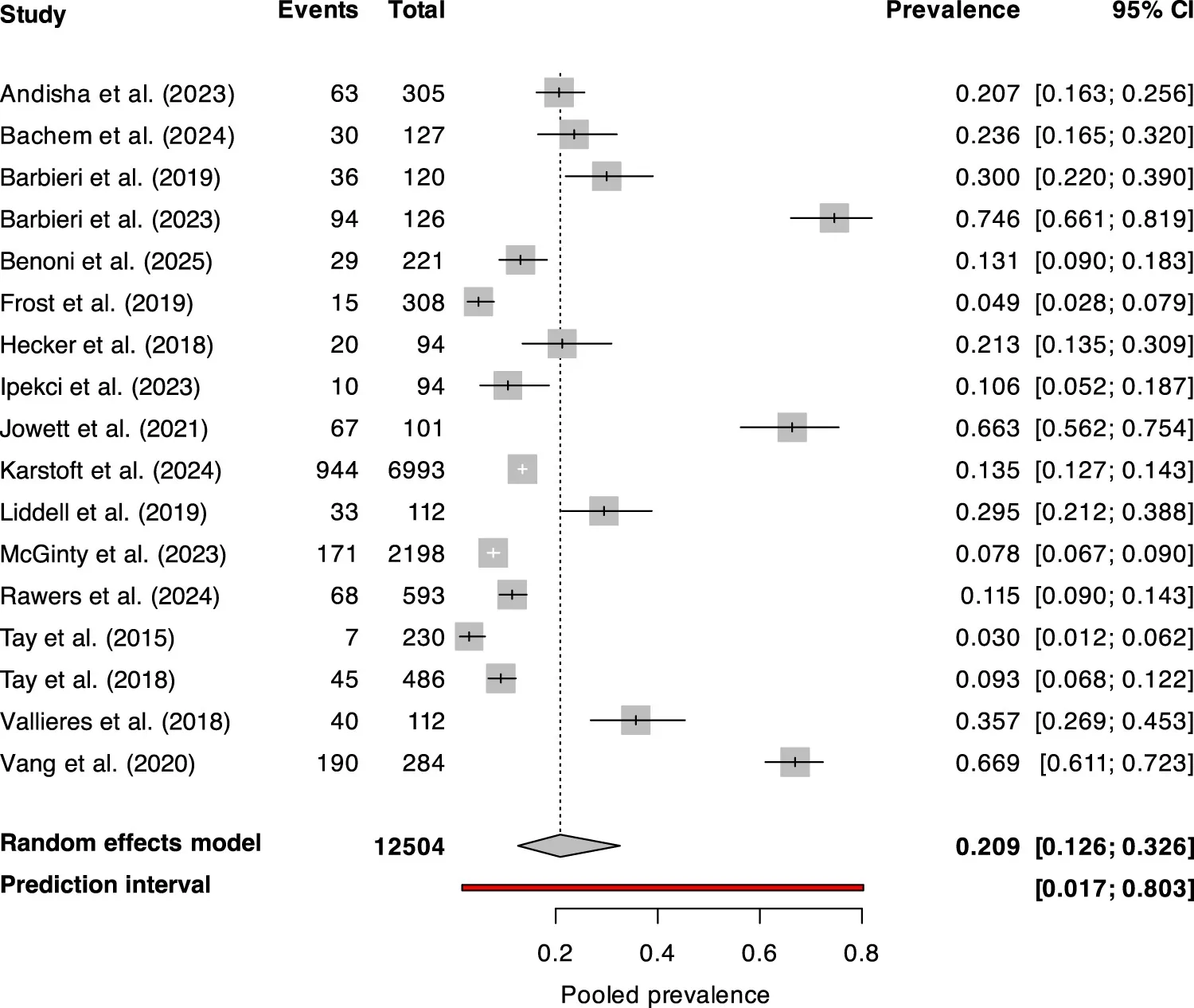
Meta-analysis of complex post-traumatic stress disorder (CPTSD) prevalence. Forest plot showing study-level prevalence estimates of CPTSD across included studies. For each study, the square represents the estimated prevalence (proportion of participants meeting CPTSD criteria), with square size proportional to the inverse-variance weight assigned in the meta-analysis. Horizontal lines indicate 95% confidence intervals (95% CI). The pooled prevalence estimate was calculated using a random-effects model, shown as a diamond, and the prediction interval reflects the expected range of prevalence estimates in comparable future studies. The x-axis represents the estimated CPTSD prevalence proportion across studies.

#### 3.4.1. Sensitivity analysis

Sequential leave-one-out sensitivity analyses revealed that exclusion of any single study did not materially alter the pooled prevalence (range: 19–23% (see Supplementary Figure 3)), indicating that the overall findings were robust. A sensitivity analysis restricting the meta-analysis to methodologically high-quality studies (JBI scores of 7–9) yielded a pooled CPTSD prevalence of 10.4% (95% CI [6.3%, 16.8%]). Substantial heterogeneity remained (*I²* = 94.0%, *τ²* = 0.44), indicating that between-study variability persisted even among studies with higher methodological quality.

#### 3.4.2. Publication bias

Visual inspection of the funnel plot indicated substantial dispersion, consistent with the high between-study heterogeneity (see Figure 3). Egger's test did not indicate significant funnel plot asymmetry (*z* = −0.85, *p* = .397). A Peters’ test, which is more appropriate for proportion data and less prone to false-positive asymmetry than Egger’s test, yielded a nominally significant result (*z* = 2.02, *p* = .044), suggesting that smaller studies may have reported higher prevalence estimates. However, given the substantial between-study heterogeneity and known differences in study populations and recruitment contexts, funnel plot asymmetry in this meta-analysis likely reflects genuine between-study variability rather than publication bias per se.

**Figure 3.**
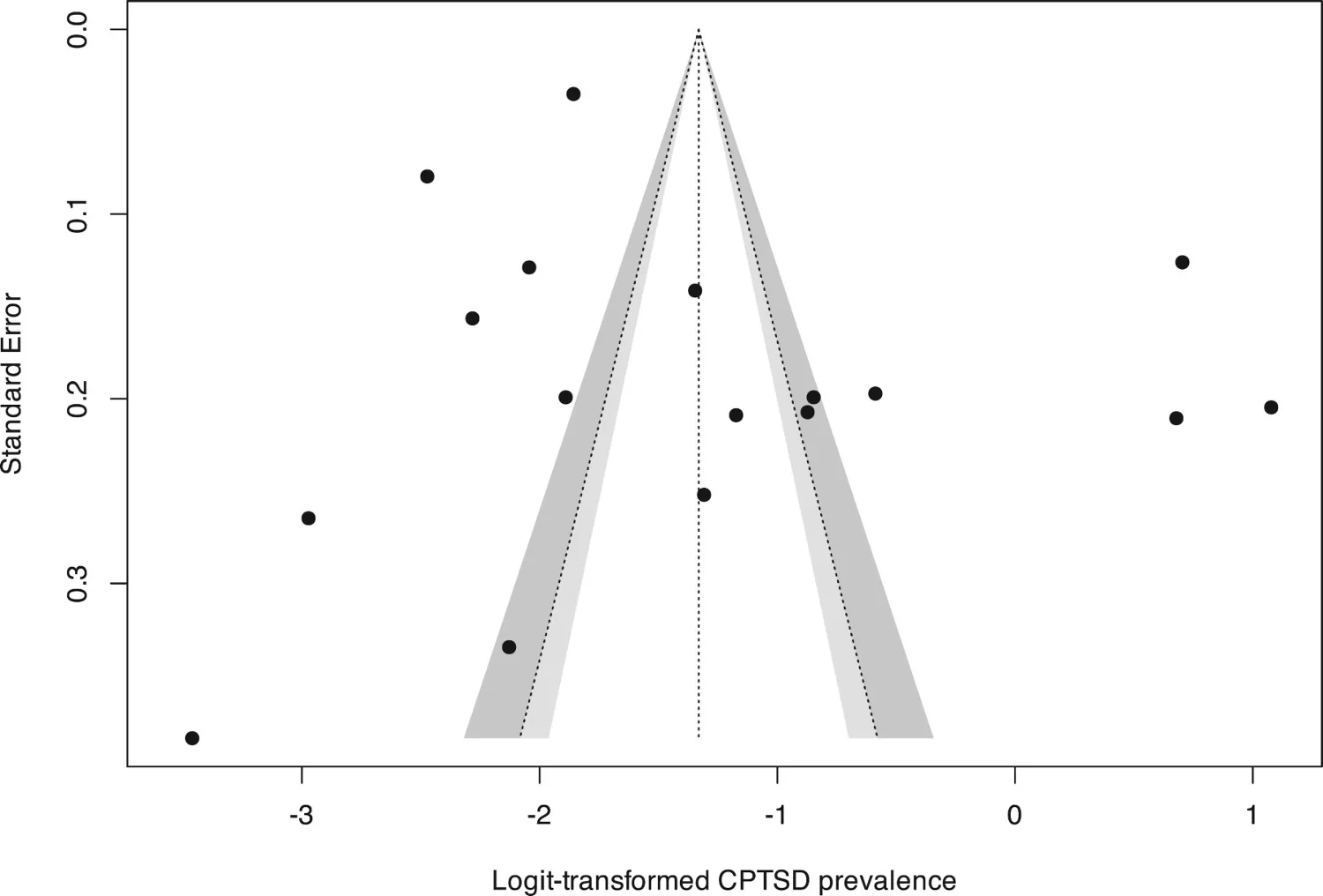
Funnel plot assessing potential publication bias. Funnel plot displaying the relationship between logit-transformed CPTSD prevalence estimates and their standard errors across included studies. Each point represents an individual study.

## 4. Discussion

This systematic review and meta-analysis synthesised evidence on the prevalence of ICD-11 CPTSD among forcibly displaced adults, including refugees, asylum seekers, and IDPs, and examined associated demographic, clinical, and trauma-related correlates. Across the included studies, prevalence ranged from 3.0% to 74.6%, and the very high between-study heterogeneity and broad prediction interval indicate that prevalence is strongly contingent on the population and context studied. Prevalence varied substantially by recruitment context and displacement subgroup, and a theoretically important pattern emerged from the narrative synthesis: post-migration living difficulties were more consistently associated with the DSO that distinguish CPTSD from PTSD than with core PTSD symptoms, suggesting that ongoing post-displacement adversity actively perpetuates complex trauma psychopathology. Between-study heterogeneity was substantial and constitutes the primary constraint on the interpretability of the pooled estimate.

The findings speak directly to the added value of the ICD-11 CPTSD construct in this population. First, CPTSD was identifiable in every setting studied and was the majority presentation in treatment-seeking samples, indicating that DSO is a common, rather than exceptional, feature of trauma-related presentations among displaced people. Second, the narrative synthesis showed that DSO symptoms were more consistently associated with post-migration living difficulties, whereas core PTSD symptoms tracked pre-migration trauma exposure. A PTSD-only framework would have missed this pattern entirely. It suggests that the ‘complex’ component of the disorder is not simply a residue of past trauma but is maintained by current legal, economic, and social conditions, which are potentially modifiable. Third, the DSO cluster carries implications for assessment and formulation, namely routine attention to shame, negative self-concept, and relational disturbance rather than re-experiencing and avoidance alone, and for treatment research: DSO symptoms have shown smaller responses to standard trauma-focused treatments in non-displaced samples (Karatzias et al., 2019a), but whether this holds for displaced populations, who have responded well to trauma-focused therapies for PTSD, remains untested.

The pooled prevalence of 20.9% is consistent with the ICD-11 theoretical framework positioning prolonged, repeated trauma from which escape is difficult or impossible, the defining exposure profile of forced displacement, as conferring heightened risk for DSO (Maercker et al., 2013). The sole prior systematic review examining CPTSD in refugees and asylum seekers identified 15 studies but reported only a narrative prevalence range of 16% to 38% in treatment-seeking samples and 2.2% to 9.3% in community-based samples for ICD-11 CPTSD, ICD-10 EPCACE, and DSM-IV DESNOS separately (de Silva et al., 2021). Notably, while de Silva et al. (2021) carefully disaggregated these estimates by diagnostic construct, the broad inclusion criteria across distinct diagnostic systems introduced conceptual diversity into the broader synthesis. EPCACE requires enduring personality change lasting at least two years (Heide et al., 2016), and DESNOS, which was never adopted as a standalone diagnosis (Mellor et al., 2021), encompasses additional domains including dissociation and somatisation absent from the ICD-11 framework. The present review builds upon this work by narrowing the focus exclusively to ICD-11-compatible instruments, providing the first pooled prevalence estimate specifically for ICD-11 CPTSD in forcibly displaced populations.

Similar to the review by de Silva et al. (2021), the most clinically important finding in our analysis was recruitment context. Treatment-seeking samples yielded a pooled prevalence of 55.2%, nearly fivefold higher than the 10.6% observed in community-based samples. This pattern is consistent with Berkson bias: specialist trauma services are self-selected for the most severe and complex presentations, such that CPTSD is the majority diagnosis in clinical settings serving displaced people rather than an edge case. This has direct implications for service design. Recognising the distinct DSO symptom cluster provides substantive clinical utility across case formulation, assessment, and treatment planning. In complex trauma, the symptoms of DSO cannot be adequately addressed through a standard PTSD framework alone. DSO requires an expanded interpretive framework that accounts for systemic regulatory dysfunction, thereby capturing the pervasive self-concept distortions and emotional instability characteristic of ICD-11 CPTSD. Standard first-line PTSD interventions primarily target re-experiencing and avoidance. However, individuals with prominent DSO features often require phased or tailored approaches that address emotion dysregulation, pervasive shame or guilt, and repair disrupted relational functioning alongside trauma processing. Although there is strong empirical support for trauma-focused interventions in treating PTSD among forcibly displaced populations, the majority of existing treatment studies were conducted before the approval of ICD-11 criteria and did not evaluate CPTSD separately (Nosè et al., 2017). Currently, direct treatment research specifically involving forcibly displaced populations diagnosed with ICD-11 CPTSD remains very limited. Phase-based approaches addressing stabilisation, affect regulation, and interpersonal difficulties, consistent with International Society for Traumatic Stress Studies (ISTSS) guidelines, are often discussed for complex presentations (Cloitre et al., 2012). However, whether tailored or phase-based strategies offer incremental benefit over standard trauma-focused protocols in this specific group remains an empirical question requiring dedicated trial evidence. Although the available community-based studies cannot support extrapolation to the global displaced population, the consistency with which CPTSD prevalence was identified across multiple settings indicates an important need for accessible assessment and care.

A theoretically important pattern emerged from the narrative synthesis. Across four studies, post-migration living difficulties were more consistently associated with DSO than PTSD symptom clusters, whereas cumulative trauma exposure, particularly war-related events and interpersonal violence, demonstrated a more robust association with core PTSD symptoms (Hecker et al., 2018; Jowett et al., 2021; Karstoft et al., 2025; Tay et al., 2018). This dissociation suggests that the features distinguishing CPTSD from PTSD may be specifically driven by chronic post-displacement adversity context rather than pre-migration trauma alone. Factors such as legal insecurity, poverty, and systemic marginalisation appear to be potent drivers of this process (Andisha & Lueger-Schuster, 2024; Jannesari et al., 2020). The clinical implication is important: CPTSD among displaced people is not simply a function of past trauma that remits once safety is reached. Instead, ongoing post-migration living difficulties may actively perpetuate DSO symptoms (Schiess-Jokanovic et al., 2021), meaning effective treatment must address current structural stressors alongside trauma processing.

Prevalence differed significantly by displacement subgroup, with asylum seekers exhibiting the highest pooled rate at 54.8%. This likely reflects the compounding burden of ongoing legal insecurity, restricted employment rights, detention conditions, and chronic inability to plan for the future, post-migration stressors constituting a form of secondary traumatisation distinct from pre-migration trauma (Gleeson et al., 2020; Li et al., 2016; Tay & Silove, 2017). However, this finding needs to be interpreted with caution: the asylum seeker subgroup comprised only three studies, two of which were treatment-seeking samples, meaning that displacement status and clinical selection effects are partially confounded. For IDPs, a single study precludes any reliable estimate (McGinty et al., 2023). Notably, IDPs may retain access to familiar social networks and cultural support by remaining within their country of origin, which could plausibly attenuate CPTSD risk relative to international refugees, though this remains speculative. Regardless, the near-absence of IDP-focused research represents a significant gap, particularly given that IDPs constitute the largest displaced subgroup globally, numbering 67.8 million as of mid-2025 (United Nations High Commissioner for Refugees, 2025).

Although the difference between ITQ-based and mapped-instrument studies did not reach statistical significance, the approximately 15 percentage point difference in pooled prevalence is clinically meaningful. Instruments such as the PCL-5 (Barbieri et al., 2019), R-MHAP (Silove et al., 2017; Tay et al., 2018), AUDADIS-IV (Frost et al., 2019), and PSS-I (Liddell et al., 2019) were designed for different diagnostic frameworks and may systematically under-measure DSO symptoms, the defining clinical distinction between PTSD and CPTSD, when mapped onto ICD-11 criteria. The ITQ, purpose-built for the ICD-11 two-dimensional diagnostic structure, requires independent threshold attainment for both PTSD symptom clusters and DSO clusters (Cloitre et al., 2018), a structural requirement that mapped instruments may fail to replicate faithfully (Hyland et al., 2017). The availability of validated ITQ adaptations in Arabic (Hyland et al., 2021), Dari (Andisha et al., 2023) and Danish (Nielsen et al., 2023) supports its feasibility across the cultural contexts most relevant to this population.

Meta-regression analyses indicated that none of the three pre-specified continuous moderators, i.e. time since displacement, mean age, or proportion of female participants, predicted CPTSD prevalence. The lack of an association with time since displacement does not imply that prolonged displacement is clinically irrelevant. At the study level, time since displacement conflates vastly different displacement trajectories, ranging from ∼10 months in recently displaced Ukrainian samples (Benoni et al., 2025; Karstoft et al., 2024; Karstoft et al., 2025; McGinty et al., 2023) to over 27 years among West Papuan refugees in protracted displacement (Silove et al., 2017; Tay et al., 2015), without accounting for resettlement stability, access to services, or specific trauma profiles. The null sex effect reflects a fundamental limitation of ecological inference: the proportion of female participants within a study is not equivalent to female sex as an individual-level predictor, and the absence of a study-level association should not be interpreted as evidence that sex does not moderate CPTSD risk. Individual-level studies consistently identify female sex as a risk factor for CPTSD (Frost et al., 2019; Liddell et al., 2019), and this finding is likely obscured at the aggregate level. Similarly, the null effects observed for age at the aggregate level likely mask significant individual-level variation.

Between-study heterogeneity was substantial and represents the principal limitation on the interpretability of the pooled estimate. The 95% prediction interval, ranging from approximately 2% to 80%, reflects the degree to which true prevalence in any given study is contingent on recruitment context, displacement subgroup, geographic and cultural setting, measurement instrument, trauma type, cumulative trauma load, time since displacement, and host-country structural conditions. Heterogeneity of this magnitude is common in prevalence meta-analyses of complex psychopathologies across diverse populations (Steel et al., 2009; Turrini et al., 2017), and the pooled point estimate is best understood as a broad central tendency rather than a precise epidemiological constant. More fundamentally, the included studies do not constitute a probability sample of the global forcibly displaced population. Geographic coverage was uneven, internally displaced persons were represented by only one independent sample, and treatment-seeking samples were disproportionately represented among some displacement subgroups. Consequently, neither the overall random-effects mean nor the subgroup means can be interpreted as globally representative population prevalence estimates.

Moreover, it is important to note that forced displacement is globally concentrated: at mid-2025, two-thirds of the world’s refugees originated from five countries, Afghanistan, Syria, Sudan, Ukraine, and Venezuela, and Sudan was the largest internal displacement situation (United Nations High Commissioner for Refugees, 2025). The studies included here map only partially onto this distribution. Syrian, Ukrainian, and Afghan samples were well represented, but Sudanese participants appeared in only one treatment-seeking sample in Niger, with Venezuelan participants were not represented at all. The Democratic Republic of the Congo, South Sudan, Myanmar, and Somalia were absent, and the only internally displaced sample was Ukrainian. Conversely, displaced Ukrainians, who represent roughly one in 20 forcibly displaced people worldwide, contributed four of the 20 studies and approximately three-quarters of all participants. This imbalance reflects where ICD-11 CPTSD research has been feasible rather than where displacement is greatest: large registry-linked surveys have been possible for Ukrainians resettled in Europe, whereas populations displaced within or across sub-Saharan Africa, Latin America, and South and Southeast Asia are rarely studied with ICD-11 instruments and, when they are, in small treatment-seeking samples. As a consequence, no inference can be drawn about CPTSD prevalence in the largest displacement situations of the present decade, which are also the settings in which post-migration living difficulties, the factor most consistently linked to DSO symptoms in this review, are likely to be most severe. Extending ICD-11 CPTSD research to these populations is therefore urgently required for any estimate that could inform global service planning.

Several additional limitations warrant acknowledgement. First, all included studies relied on self-report instruments, introducing the possibility of over- or under-reporting of symptom severity. Second, the predominantly cross-sectional design of included studies precludes causal inference regarding the temporal relationship between post-migration stressors and CPTSD. Third, restriction to English-language publications may have introduced geographic publication bias, potentially underrepresenting evidence from non-Western contexts. Fourth, translation procedures, cultural adaptation, psychometric evaluation, and population-specific validation of the CPTSD instruments were frequently under-reported or absent. Only 10 of the 20 studies used an instrument validated in the language and population studied; the remainder had no identifiable validation history in the relevant population. Psychometric evidence in the study sample was reported by 15 studies but was predominantly limited to internal consistency. Only one study reported reliability at the level of individual symptom clusters, and confirmatory factor analysis was reported for only five independent samples. Notably, the three studies that used an instrument developed and culturally adapted for the population studied (West Papuan refugees) did not support the ICD-11 hierarchical PTSD/DSO structure, so that even where validated translations exist, the conceptual equivalence of DSO constructs across cultural contexts cannot be assumed and warrants further psychometric investigation. These gaps introduce potential measurement bias that should be borne in mind when interpreting the pooled and subgroup estimates, although prevalence did not differ significantly between studies with and without validated instruments (18.6% vs. 23.1%).

Our findings carry direct clinical and policy implications. Given that approximately one in ten forcibly displaced adults in community settings meets CPTSD criteria, rising to more than one in two in treatment-seeking settings, routine screening for CPTSD is warranted across health services for displaced people, with the ITQ as the recommended instrument. The high prevalence of CPTSD in forcibly displaced populations necessitates practical, scalable responses that extend beyond specialist clinical care: reducing bureaucratic delays in legal status determination, ensuring access to stable housing and employment, and strengthening community-based social support, the structural conditions that the evidence suggests directly drive DSO symptom burden. Task-sharing models, in which trained lay workers or community health workers deliver structured psychosocial support, offer a feasible pathway to addressing CPTSD at scale in resource-limited humanitarian settings (Bryant et al., 2023). The disproportionate burden among asylum seekers underscores the critical need to deliver mental health support before, not only after, refugee status determination. At the policy level, extrapolating the community prevalence to the current scale of global displacement implies tens of millions of people with unmet CPTSD treatment needs, requiring strategic investment in these models, stepped-care approaches, and culturally adapted interventions.

Several priorities for future research emerge from this review. Individual participant data meta-analyses are required to examine individual-level predictors, particularly sex, age, trauma type, post-migration living difficulties, and their interaction, which cannot be adequately addressed at the aggregate study level. Longitudinal studies are essential to map the natural course of CPTSD in displaced populations, including rates of remission, the impact of resettlement conditions, and predictors of chronicity. Despite constituting the largest displaced subgroup globally, IDPs remain severely under-studied and represent an urgent research priority. Current evidence is also heavily concentrated on Middle Eastern, African, and Ukrainian or European contexts, with studies in under-represented regions such as Latin America, East and Southeast Asia, and the Pacific remaining scarce. Future prevalence studies should standardise the reporting of post-migration living difficulties, trauma load, and time since displacement to enhance the comparability and statistical power of subsequent meta-analyses. Finally, RCTs evaluating CPTSD-specific treatments within displaced populations are urgently needed to translate this epidemiological evidence into clinical practice (Hassija & Cloitre, 2013; Schäfer et al., 2022).

This systematic review and meta-analysis provides the first quantitative synthesis of ICD-11 CPTSD prevalence rates among forcibly displaced adults. Across the included studies, roughly one in five participants met diagnostic criteria, but the evidence demonstrates pronounced variation across populations and contexts, and no single estimate can be generalised to the global forcibly displaced population. Recruitment setting accounted for an important component of this variation, with substantially higher prevalence in treatment-seeking samples and among asylum seekers, underscoring the compounding effect of ongoing legal and structural adversity on complex trauma psychopathology. A consistent pattern across studies links post-migration living difficulties specifically to DSO symptoms, suggesting that CPTSD in this population is actively maintained by post-displacement structural conditions rather than past trauma alone. These findings affirm the added value of the ICD-11 CPTSD construct in this population: DSO symptoms are common, are linked to modifiable post-migration conditions, and reinforce the need for ICD-11-informed screening, treatment research that evaluates CPTSD-specific outcomes, and socially informed care. Given that global displacement now exceeds 117 million individuals (United Nations High Commissioner for Refugees, 2025), addressing the CPTSD burden in this population is a public health imperative.

## Supplementary Material

Supplementary_Materials.pdf

## Data Availability

This study is a systematic review and meta-analysis based entirely on previously published studies. No new data were collected or generated. All data used in this review are available in the original published sources cited herein.
