# Supplementary material for: Complex post-traumatic stress disorder in refugees, asylum seekers, and internally displaced people: a systematic review and meta-analysis
Source: Eur J Psychotraumatol. 2026 Oct 20;17(1):2735202. doi: 10.1080/20008066.2026.2735202 (PMC13686194; doi:10.1080/20008066.2026.2735202)
Supplement: Supplementary file 1 [file ZEPT_A_2735202_SM8460.pdf]

Supplementary Materials

Table of Contents

Supplemental Table 1: Preferred Reporting Items for Systematic Review and Meta-Analysis (PRISMA) 2020 guidelines. 2

Supplementary Table 2A: Critical Appraisal Checklist for Observational Studies of included articles by the Joanna Briggs Institute (JBI). 5

Supplementary Table 2B. Appraisal of CPTSD instrument validity in the included studies (expanded JBI item 6) 6

Supplementary Figure 1. Subgroup meta-analysis of complex post-traumatic stress disorder (CPTSD) prevalence by measurement approach. 10

Supplementary Figure 2. Subgroup meta-analysis of complex post-traumatic stress disorder (CPTSD) prevalence by recruitment source. 11

Supplementary Figure 3. Leave-one-out sensitivity analysis of complex post-traumatic stress disorder (CPTSD) prevalence. 13

Supplementary Figure 4. Subgroup meta-analysis of complex post-traumatic stress disorder (CPTSD) prevalence by income classification of the country of resettlement. 14

Supplementary Figure 5. Subgroup meta-analysis of complex post-traumatic stress disorder (CPTSD) prevalence by validation of the measurement tools. 15

Supplemental Table 1: Preferred Reporting Items for Systematic Review and Meta-Analysis (PRISMA) 2020 guidelines.

Section and Topic		Checklist item	Location where item is reported
**TITLE: Complex Post-Traumatic Stress Disorder in Internally Displaced People, Asylum Seekers and Refugees: A Systematic Review and Meta-Analysis**			
Title	1	Identify the report as a systematic review.	1
**ABSTRACT**			
Abstract	2	See the PRISMA 2020 for Abstracts checklist.	2
**INTRODUCTION**			
Rationale	3	Describe the rationale for the review in the context of existing knowledge.	3-4
Objectives	4	Provide an explicit statement of the objective(s) or question(s) the review addresses.	4
**METHODS**			
Eligibility criteria	5	Specify the inclusion and exclusion criteria for the review and how studies were grouped for the syntheses.	5-6
Information sources	6	Specify all databases, registers, websites, organisations, reference lists and other sources searched or consulted to identify studies. Specify the date when each source was last searched or consulted.	5
Search strategy	7	Present the full search strategies for all databases, registers and websites, including any filters and limits used.	5
Selection process	8	Specify the methods used to decide whether a study met the inclusion criteria of the review, including how many reviewers screened each record and each report retrieved, whether they worked independently, and if applicable, details of automation tools used in the process.	6
Data collection process	9	Specify the methods used to collect data from reports, including how many reviewers collected data from each report, whether they worked independently, any processes for obtaining or confirming data from study investigators, and if applicable, details of automation tools used in the process.	6-7
Data items	10a	List and define all outcomes for which data were sought. Specify whether all results that were compatible with each outcome domain in each study were sought (e.g. for all measures, time points, analyses), and if not, the methods used to decide which results to collect.	7-8
	10b	List and define all other variables for which data were sought (e.g. participant and intervention characteristics, funding sources). Describe any assumptions made about any missing or unclear information.	NA
Study risk of bias assessment	11	Specify the methods used to assess risk of bias in the included studies, including details of the tool(s) used, how many reviewers assessed each study and whether they worked independently, and if applicable, details of automation tools used in the process.	7
Effect measures	12	Specify for each outcome the effect measure(s) (e.g. risk ratio, mean difference) used in the synthesis or presentation of results.	NA
Synthesis methods	13a	Describe the processes used to decide which studies were eligible for each synthesis (e.g. tabulating the study intervention characteristics and comparing against the planned groups for each synthesis (item #5)).	5-6
	13b	Describe any methods required to prepare the data for presentation or synthesis, such as handling of missing summary statistics, or data conversions.	7-8
	13c	Describe any methods used to tabulate or visually display results of individual studies and syntheses.	8
	13d	Describe any methods used to synthesize results and provide a rationale for the choice(s). If meta-analysis was performed, describe the model(s), method(s) to identify the presence and extent of statistical heterogeneity, and software package(s) used.	7
	13e	Describe any methods used to explore possible causes of heterogeneity among study results (e.g. subgroup analysis, meta-regression).	7-8
	13f	Describe any sensitivity analyses conducted to assess robustness of the synthesized results.	7-8
Reporting bias assessment	14	Describe any methods used to assess risk of bias due to missing results in a synthesis (arising from reporting biases).	8

Section and Topic		Checklist item	Location where item is reported
Certainty assessment	15	Describe any methods used to assess certainty (or confidence) in the body of evidence for an outcome.	7
**RESULTS**			
Study selection	16a	Describe the results of the search and selection process, from the number of records identified in the search to the number of studies included in the review, ideally using a flow diagram.	8
	16b	Cite studies that might appear to meet the inclusion criteria, but which were excluded, and explain why they were excluded.	8
Study characteristics	17	Cite each included study and present its characteristics.	8-9
Risk of bias in studies	18	Present assessments of risk of bias for each included study.	12
Results of individual studies	19	For all outcomes, present, for each study: (a) summary statistics for each group (where appropriate) and (b) an effect estimates and its precision (e.g. confidence/credible interval), ideally using structured tables or plots.	12-13
Results of syntheses	20a	For each synthesis, briefly summarise the characteristics and risk of bias among contributing studies.	12
	20b	Present results of all statistical syntheses conducted. If meta-analysis was done, present for each the summary estimate and its precision (e.g. confidence/credible interval) and measures of statistical heterogeneity. If comparing groups, describe the direction of the effect.	12-13
	20c	Present results of all investigations of possible causes of heterogeneity among study results.	12-13
	20d	Present results of all sensitivity analyses conducted to assess the robustness of the synthesized results.	13
Reporting biases	21	Present assessments of risk of bias due to missing results (arising from reporting biases) for each synthesis assessed.	12
Certainty of evidence	22	Present assessments of certainty (or confidence) in the body of evidence for each outcome assessed.	13
**DISCUSSION**			
Discussion	23a	Provide a general interpretation of the results in the context of other evidence.	14
	23b	Discuss any limitations of the evidence included in the review.	17
	23c	Discuss any limitations of the review processes used.	17
	23d	Discuss implications of the results for practice, policy, and future research.	17
**OTHER INFORMATION**			
Registration and protocol	24a	Provide registration information for the review, including register name and registration number, or state that the review was not registered.	4
	24b	Indicate where the review protocol can be accessed, or state that a protocol was not prepared.	4
	24c	Describe and explain any amendments to information provided at registration or in the protocol.	4
Support	25	Describe sources of financial or non-financial support for the review, and the role of the funders or sponsors in the review.	19
Competing interests	26	Declare any competing interests of review authors.	19
Availability of	27	Report which of the following are publicly available and where they can be found template data collection forms; data extracted from included	19

data, code and other materials		studies; data used for all analyses; analytic code; any other materials used in the review.	19

From: Page MJ, McKenzie JE, Bossuyt PM, Boutron I, Hoffmann TC, Mulrow CD, et al. The PRISMA 2020 statement: an updated guideline for reporting systematic reviews. BMJ 2021;372:n71. doi: 10.1136/bmj.n71. This work is licensed under CC BY 4.0. To view a copy of this license, visit https://creativecommons.org/licenses/by/4.0/

Supplementary Table 2A: Critical Appraisal Checklist for Observational Studies of included articles by the Joanna Briggs Institute (JBI).

**Authors/ publication year**	**Q1**	**Q2**	**Q3**	**Q4**	**Q5**	**Q6**	**Q7**	**Q8**	**Q9**	**Total**	**Risk of bias**
Andisha et al., 2023	1	0	0	1	1	1	0	1	0	5	Moderate risk of bias
Bachem et al., 2024	1	0	1	1	1	1	1	1	0	7	Low risk of bias
Barbieri et al., 2019	1	0	0	1	1	1	0	1	0	5	Moderate risk of bias
Barbieri et al., 2023	1	0	0	1	1	1	1	1	0	6	Moderate risk of bias
Benoni et al, 2025	1	0	1	1	1	1	1	1	0	7	Low risk of bias
Frost et al., 2019	1	1	0	1	0	0	0	0	1	4	Moderate risk of bias
Hecker et al., 2018	1	0	0	1	1	1	0	1	0	5	Moderate risk of bias
Hyland et al., 2018	1	0	0	1	1	1	1	1	0	6	Moderate risk of bias
Ipekci et al., 2023	0	0	0	1	1	1	1	1	0	5	Moderate risk of bias
Jowett et al., 2021	1	0	0	1	0	1	0	1	0	4	Moderate risk of bias
Karstoft et al, 2024	1	1	1	1	1	1	1	1	1	9	Low risk of bias
Karstoft et al., 2025	1	1	1	1	1	1	1	1	1	9	Low risk of bias
Liddell et al, 2019	1	0	0	1	1	0	0	1	0	4	Moderate risk of bias
McGinty et al., 2023	1	1	1	1	1	1	1	1	1	9	Low risk of bias
Rawers et al., 2024	1	0	1	0	1	1	1	1	0	6	Moderate risk of bias
Silove et al., 2017	1	1	1	1	1	0	1	1	1	8	Low risk of bias
Tay et al., 2015	1	1	1	1	1	0	1	1	1	8	Low risk of bias
Tay et al., 2018	1	1	1	1	1	0	1	1	1	8	Low risk of bias
Vallièeres et al., 2018	1	0	0	1	1	0	1	1	0	5	Moderate risk of bias
Vang et al., 2020	1	0	0	1	1	1	1	1	0	6	Moderate risk of bias

Supplementary Table 2B. Appraisal of CPTSD instrument validity in the included studies (expanded JBI item 6)

Study	Instrument and language(s) of administration	Translation / cultural adaptation reported	Prior validation of the version administered	Psychometric evidence in the study sample	Factor analysis of the instrument in the study sample	JBI item 6[b]
Andisha et al. (2023)	ITQ<br>**Language:** Dari	**Yes:** Beaton guidelines; two forward translations, blind back-translation, pilot (n = 30)	**Validated in language:** this study<br>**Validated in displaced population:** this study (Afghan refugees)	**α:** PTSD = .83, DSO = .88, total = .90<br>**CR:** PTSD = .88, DSO = .93	**CFA (MLR):** two-factor second-order model best fitting<br>**Fit:** CFI = .965, TLI = .950, RMSEA = .056 [.038–.073], SRMR = .045	1
Bachem et al. (2024)	ITQ<br>**Language:** Tigrinya	**Yes:** forward + blind back-translation, consensus, pilot	**Validated in language:** no<br>**Validated in displaced population:** no (authors state not validated)	**α:** PTSD = .86, DSO = .94	None	0
Barbieri et al. (2019)	PCL-5 items mapped to ICD-11 (13 items)<br>**Languages:** English, French, Arabic (read aloud with cultural mediator)	**No:** existing PCL-5 versions	**Validated in language:** PCL-5 yes; ICD-11 mapping no<br>**Validated in displaced population:** no (authors: DSO items from a scale not validated for the construct)	**α:** PCL-5 total = .87 [.84–.90]; mapped 13-item subset = .81 [.76–.86]<br>**PTSD/DSO subscales:** not reported	None (LCA only)	0
Barbieri et al. (2023)	ITQ<br>**Languages:** Arabic, English, French (read aloud; 86% Sudanese, mainly Arabic)	Not described ("translated versions read out loud")	**Validated in language:** Arabic yes (Vallières et al., 2018); English/French no<br>**Validated in displaced population:** Arabic yes	**α:** PTSD = .70, DSO = .86, total = .87; functional impairment = .88	None (LCA only)	1[a]
Benoni et al. (2025)	ITQ<br>**Languages:** Ukrainian, Russian	**No:** existing versions	**Validated in language:** yes (Karatzias et al., 2023, parents in Ukraine)<br>**Validated in displaced population:** no	**α:** PTSD = .88 [.85–.90]; CPTSD scale = .88 [.86–.91]	None	0
Frost et al. (2019)	AUDADIS-IV interview items mapped to ICD-11 (dichotomous; no functional-impairment criterion)<br>**Language:** English	Not applicable	**Validated in language:** not applicable<br>**Validated in displaced population:** no	None in sample (cites AUDADIS test–retest reliability in the general population)	**CFA of mapped items:** correlated six-factor model<br>**Fit:** CFI = 1.00, TLI = 1.005, RMSEA = .00	0

Study	Instrument and language(s) of administration	Translation / cultural adaptation reported	Prior validation of the version administered	Psychometric evidence in the study sample	Factor analysis of the instrument in the study sample	JBI item 6[b]
					**FMM:** five classes	
Hecker et al. (2018)	ITQ v1.4 (7 PTSD / 16 DSO items)<br>**Languages:** written versions in English, French, German, Spanish, Arabic, Turkish, Tamil; assessed in 12 languages (Arabic 49%, Persian 17%, Tigrinya 14% via interpreter-led interview)	**Yes:** native/trained translators + blind back-translation to English	**Validated in language:** partial (Arabic yes)<br>**Validated in displaced population:** partial; authors note not validated within each cultural group	**α:** PTSD = .88, DSO = .85	None	0[a]
Hyland et al. (2018)	ITQ v1.3 (6 PTSD / 16 DSO items)<br>**Language:** Arabic	**Yes:** professional translation + back-translation	**Validated in language:** yes<br>**Validated in displaced population:** yes (Vallières et al., 2018, same sample)	**α:** PTSD = .76, DSO = .88; functional impairment = .82	None (LCA only; CFA reported in Vallières et al., 2018)	1
Ipekci (2023)	ITQ<br>**Language:** Arabic (existing version)	No	**Validated in language:** yes<br>**Validated in displaced population:** yes (Vallières et al., 2018)	Not reported for this sample (α values cited are from Hyland et al., 2018)	None	1
Jowett et al. (2021)	ITQ<br>**Language:** English, administered through interpreters (languages not specified)	No	**Validated in language:** no<br>**Validated in displaced population:** no	None (service evaluation; 23.8% ITQ data missing)	None	0
Karstoft et al. (2024)	ITQ<br>**Languages:** Ukrainian, Russian (official versions)	No new translation; focus group (n = 10) and pilot (n = 97)	**Validated in language:** yes (Karatzias et al., 2023)<br>**Validated in displaced population:** no	Not reported	None	0
Karstoft et al. (2025)	ITQ<br>**Languages:** Ukrainian, Russian	No	**Validated in language:** yes (Karatzias et al., 2023)<br>**Validated in displaced population:** no	Not reported	None	0

Study	Instrument and language(s) of administration	Translation / cultural adaptation reported	Prior validation of the version administered	Psychometric evidence in the study sample	Factor analysis of the instrument in the study sample	JBI item 6[b]
Liddell et al. (2019)	PSS-I items (PTSD, 6 items) + ITQ precursor (DSO; 9-item version n = 94, 17-item version n = 16)<br>**Language:** English with interpreter support	No	**Validated in language:** no<br>**Validated in displaced population:** no	**α:** PTSD (PSS-I) = .80; DSO = .75 (9-item) / .92 (17-item)	None (LCA only)	0
McGinty et al. (2023)	Pre-finalised ITQ (12 core items)<br>**Languages:** Ukrainian/Russian	No	**Validated in language:** yes (Karatzias et al., 2023)<br>**Validated in displaced population:** no	**α:** total 12-item scale = .89<br>**PTSD/DSO subscales:** not reported	None for the ITQ (factor analysis in the paper concerns the Brief COPE)	0
Rawers et al. (2024)	ITQ<br>**Language:** Arabic (existing version)	No	**Validated in language:** yes<br>**Validated in displaced population:** yes (Vallières et al., 2018)	**α:** PTSD = .89, DSO = .90	None (LCA of trauma exposure only)	1
Silove et al. (2017)	R-MHAP culturally adapted module (mapped ICD-11 items, dichotomous; interviewer-administered)<br>**Language:** Bahasa Indonesian	**Cultural adaptation:** focus groups (n = 20) and PNG psychiatrists; indigenous symptom terms documented<br>**Formal translation:** not described	**Validated in language:** yes<br>**Validated in displaced population:** yes (Tay et al., 2015, same sample)	**KR-20:** PTSD = .93, CPTSD = .88<br>**Stability:** 6-month follow-up (n = 101)<br>**Inter-rater agreement:** 90%	**CFA (WLSMV):** one-factor $\chi^2(7)$ = 7.33, CFI = 1.00, TLI = 1.00, RMSEA = .01; two-factor $\chi^2(8)$ = 9.80, CFI = 1.00, TLI = 1.00, RMSEA = .03; factor r = .95 → one-factor preferred<br>**Second-order model:** failed to converge	1
Tay et al. (2015)	R-MHAP (as above)<br>**Language:** Bahasa Indonesian	**Cultural adaptation:** as above (two rounds of focus groups)	**Validated in language:** this study<br>**Validated in displaced population:** this study	**KR-20:** PTSD item pool = .93 (T1) / .94 (T2)<br>**Test–retest:** 6-month follow-up (n = 101), no significant change<br>**Convergent validity:** r = .55 with HTQ<br>**Inter-rater agreement:** 90%	**CFA (WLSMV):** ICD-11 PTSD three-factor $\chi^2(14)$ = 3.43, CFI = .99, TLI = .99, RMSEA = .00; CPTSD six-factor $\chi^2(40)$ = 38.03, CFI = .99, TLI = .99, RMSEA = .00<br>**Second-order six-factor:** poor fit (CFI = .93, TLI = .92)	1
Tay et al. (2018)	R-MHAP<br>**Languages:** Bahasa Indonesian, English, Tok Pisin (interviewer; participant's choice)	**Cultural adaptation:** focus groups (n = 40) and psychiatrists	**Validated in language:** yes<br>**Validated in displaced population:** yes (Tay et al., 2015)	**α:** CPTSD = .95, PTSD = .97<br>**Inter-rater agreement:** 95%<br>**Convergent validity:** r = .76 with HTQ	**CFA (WLSMV):** six-factor $\chi^2(39)$ = 272.93, CFI = .95, TLI = .92, RMSEA = .08 (moderate)<br>**One-factor and higher-order models:** poor fit (RMSEA ≥ .11)	1

Study	Instrument and language(s) of administration	Translation / cultural adaptation reported	Prior validation of the version administered	Psychometric evidence in the study sample	Factor analysis of the instrument in the study sample	JBI item 6[b]
				**SCID concordance:** AUC = .93 (earlier sub-sample)		
Vallières et al. (2018)	Preliminary ITQ (6 PTSD / 16 DSO items)<br>**Language:** Arabic	**Yes:** professional translation + back-translation	**Validated in language:** this study<br>**Validated in displaced population:** this study	**CR:** PTSD = .80, DSO = .94	**CFA (WLSMV):** two-factor second-order model (affective dysregulation split hyper-/hypo-activation) best fitting; all loadings > .50; PTSD–DSO r = .88<br>**Fit: Model** 1: CFI = 0.907, TLI = 0.897, RMSEA= 0.072<br>**Modules** 2 & 3 are rejected<br>**Model 4**: CFI = 0.946, TLI = 0.938, RMSEA = 0.056<br>**Model 5**: CFI = 0.950, TLI = 0.943, RMSEA = 0.054<br>**Model 6**: CFI = 0.957, TLI = 0.950, RMSEA = 0.050	1
Vang et al. (2020)	ITQ (12 items)<br>**Languages:** Danish, Bosnian, Arabic; 70% needed an interpreter (items read aloud)	**Yes:** Danish (Elklit, 2015); Bosnian and Arabic back-translation with trained interpreters/clinicians	**Validated in language:** Danish yes, Arabic yes; Bosnian no<br>**Validated in displaced population:** Arabic yes; Danish in clinical samples	**α:** PTSD = .74, DSO = .83, total = .84<br>**Clusters:** Re = .70, Av = .62, Hyp = .55, AD = .53, NSC = .82, DR = .73	None (LCA only)	1[a]

[a] More than one language version administered; rating based on the version administered to the majority of the sample.

[b] JBI item 6 was scored 1 where the instrument, in the language administered to the majority of the sample, had been validated in a forcibly displaced population prior to or within the study, and 0 otherwise. This binary rating defined the instrument-validation subgroups in the meta-analysis (10 of 20 studies; 8 of 17 independent samples).

Sample sizes and settings are reported in Supplementary Table 2. Abbreviations: α = Cronbach's alpha; CR = composite reliability; KR-20 = Kuder–Richardson 20 (dichotomous items); ITQ = International Trauma Questionnaire; R-MHAP = Refugee Mental Health Assessment Package; PSS-I = PTSD Symptom Scale–Interview; PCL-5 = PTSD Checklist for DSM-5; AUDADIS-IV = Alcohol Use Disorder and Associated Disabilities Interview Schedule-IV; CFA = confirmatory factor analysis; LCA = latent class analysis; FMM = factor mixture modelling; HTQ = Harvard Trauma Questionnaire; SCID = Structured Clinical Interview for DSM; DSO = disturbances in self-organisation; Re/Av/Hyp/AD/NSC/DR = re-experiencing, avoidance, hypervigilance, affective dysregulation, negative self-concept, disturbed relationships. 'Not reported' = no psychometric statistics given for the study sample.

Supplementary Figure 1. Subgroup meta-analysis of complex post-traumatic stress disorder (CPTSD) prevalence by measurement approach.

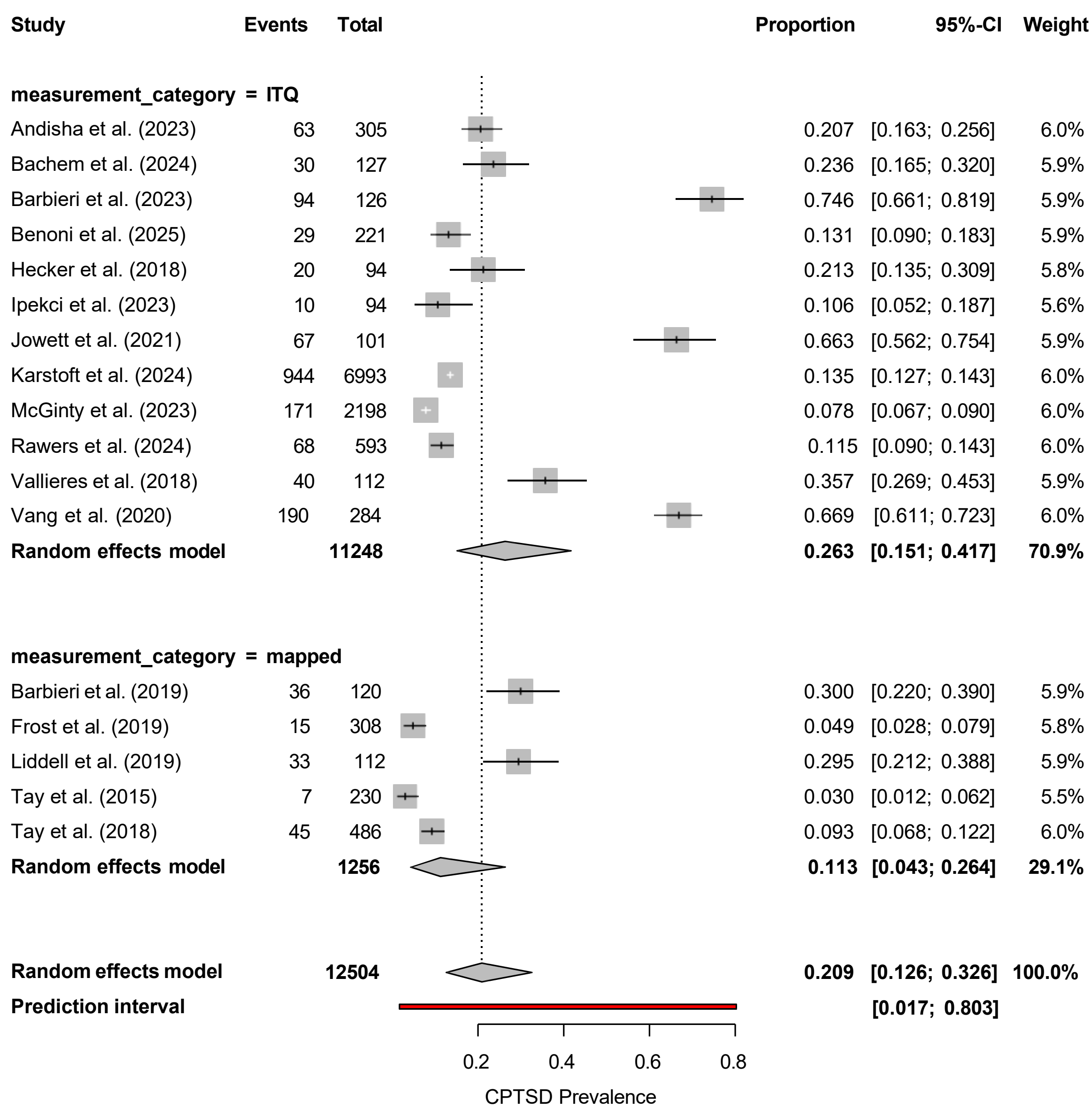

Forest plot showing study-level prevalence estimates of CPTSD across included studies, stratified by measurement category. Studies are grouped according to whether CPTSD was assessed using the International Trauma Questionnaire (ITQ) or mapped from other instruments. For each study, the square represents the estimated prevalence (proportion of participants meeting CPTSD criteria), with square size proportional to the inverse-variance weight assigned in the meta-analysis. Horizontal lines indicate 95% confidence intervals (95% CI) around each study estimate. Within each subgroup, pooled prevalence estimates were calculated using a random-effects model, shown as diamonds. The prediction interval reflects the expected range of prevalence estimates in comparable future studies. The x-axis represents the estimated CPTSD prevalence proportion.

Supplementary Figure 2. Subgroup meta-analysis of complex post-traumatic stress disorder (CPTSD) prevalence by recruitment source.

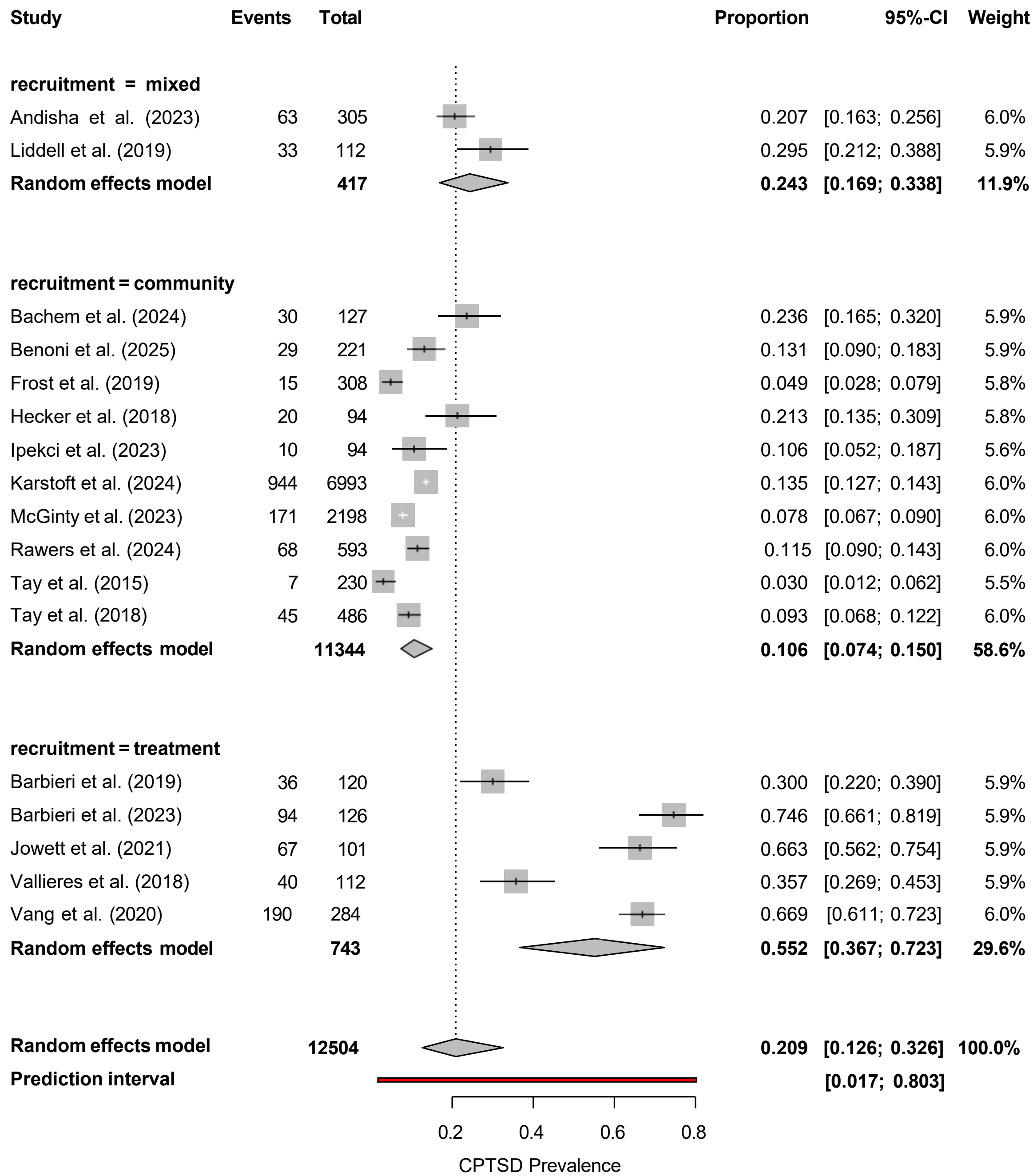

Forest plot showing study-level prevalence estimates of CPTSD across included studies, stratified by recruitment source. Studies are grouped into community, treatment, and mixed recruitment samples. For each study, the square represents the estimated prevalence (proportion of participants meeting CPTSD criteria), with square size proportional to the inverse-variance weight assigned in the meta-analysis. Horizontal lines indicate 95% confidence intervals (95% CI) for each study estimate. Within each subgroup, pooled prevalence estimates were calculated

using a random-effects model, displayed as diamonds. The prediction interval reflects the expected range of prevalence estimates in comparable future studies. The x-axis represents the estimated CPTSD prevalence proportion.

Supplementary Figure 3. Leave-one-out sensitivity analysis of complex post-traumatic stress disorder (CPTSD) prevalence.

Study	Proportion	95% CI	τ^2	I^2
Omitting Andisha et al. (2023)	0.21	[0.12; 0.33]	1.6791	98.4%
Omitting Bachem et al. (2024)	0.21	[0.12; 0.33]	1.6760	98.4%
Omitting Barbieri et al. (2019)	0.20	[0.12; 0.33]	1.6616	98.4%
Omitting Barbieri et al. (2023)	0.19	[0.12; 0.29]	1.2634	98.0%
Omitting Benoni et al. (2025)	0.21	[0.13; 0.34]	1.6546	98.4%
Omitting Frost et al. (2019)	0.23	[0.14; 0.35]	1.4871	98.4%
Omitting Hecker et al. (2018)	0.21	[0.12; 0.33]	1.6764	98.4%
Omitting Ipekci et al. (2023)	0.22	[0.13; 0.34]	1.6306	98.4%
Omitting Jowett et al. (2021)	0.19	[0.11; 0.30]	1.3908	98.1%
Omitting Karstoft et al. (2024)	0.21	[0.13; 0.34]	1.6587	98.3%
Omitting Liddell et al. (2019)	0.20	[0.12; 0.33]	1.6631	98.4%
Omitting McGinty et al. (2023)	0.22	[0.13; 0.35]	1.5802	98.2%
Omitting Rawers et al. (2024)	0.22	[0.13; 0.34]	1.6407	98.4%
Omitting Tay et al. (2015)	0.23	[0.14; 0.35]	1.3870	98.4%
Omitting Tay et al. (2018)	0.22	[0.13; 0.35]	1.6110	98.4%
Omitting Vallieres et al. (2018)	0.20	[0.12; 0.32]	1.6389	98.3%
Omitting Vang et al. (2020)	0.19	[0.11; 0.29]	1.3723	97.4%
**Random effects model**	**0.21**	**[0.13; 0.33]**	**1.5687**	**98.3%**

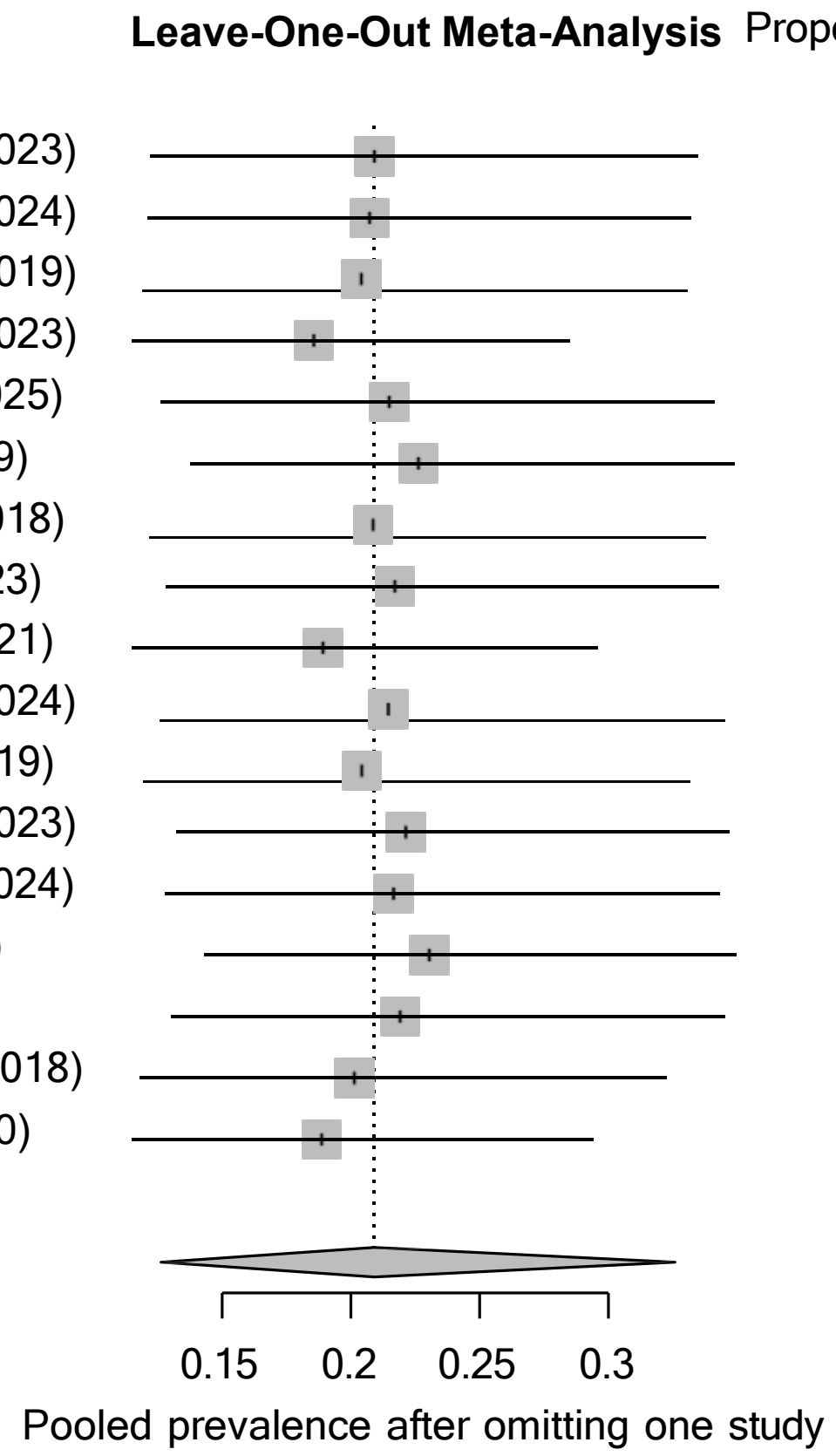

Leave-one-out meta-analysis examining the influence of individual studies on the pooled prevalence estimate of CPTSD. Each row shows the pooled prevalence calculated using a random-effects model after sequentially omitting one study from the analysis. Points represent the pooled prevalence estimate (proportion of participants meeting CPTSD criteria) after exclusion of the specified study, with horizontal lines indicating 95% confidence intervals (95% CI). The first row shows the pooled estimate including all studies. The analysis also reports between-study heterogeneity statistics for each iteration, including τ² (between-study variance) and I² (percentage of total variability attributable to heterogeneity rather than sampling error). The x-axis shows the pooled CPTSD prevalence estimate following omission of each study.

Supplementary Figure 4. Subgroup meta-analysis of complex post-traumatic stress disorder (CPTSD) prevalence by income classification of the country of resettlement.

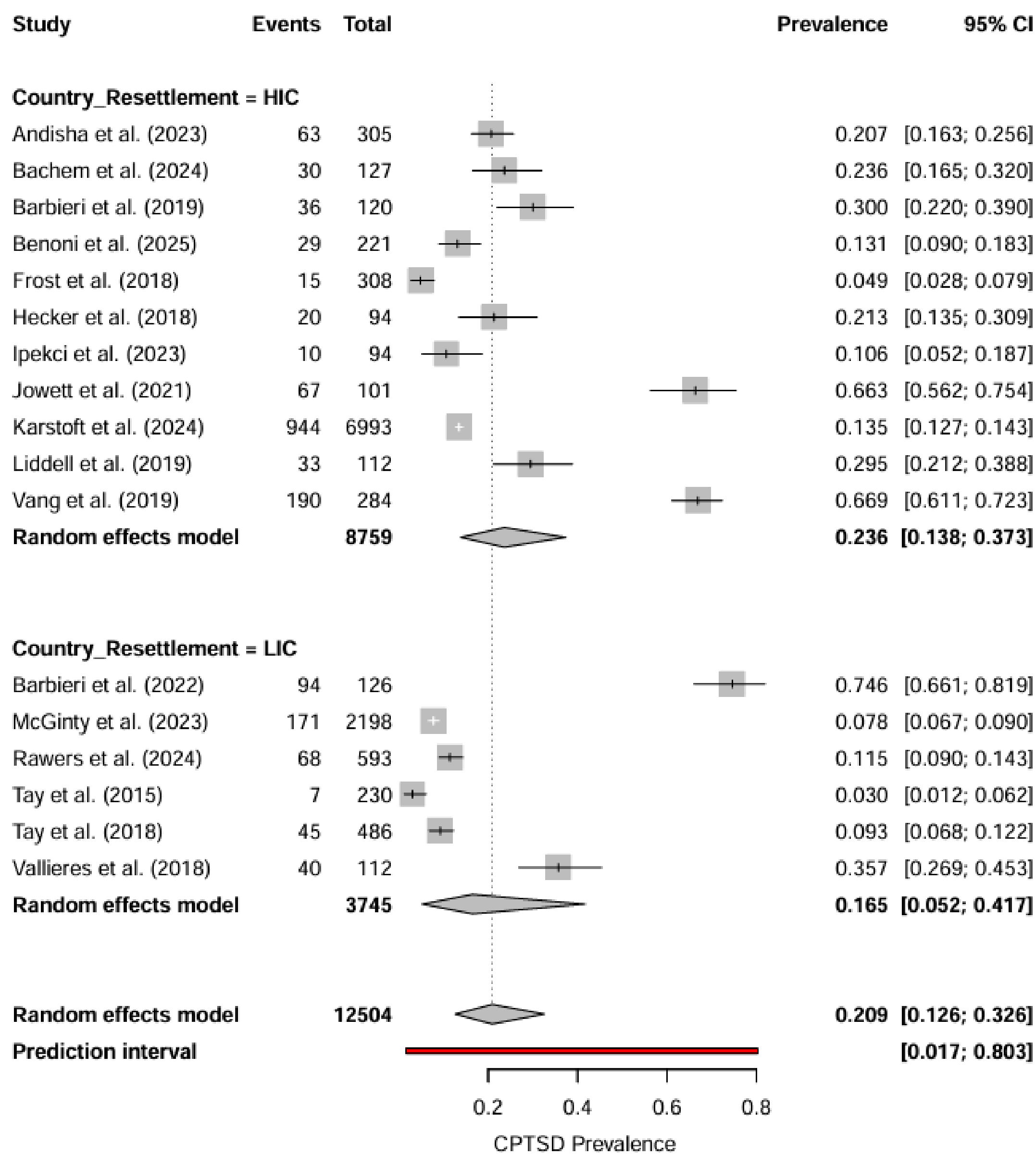

Forest plot displaying the subgroup analysis of CPTSD prevalence based on the income classification of the country of resettlement (high-income versus low- and middle-income countries) among forcibly displaced populations. For each study, the square represents the estimated prevalence (proportion of participants meeting CPTSD criteria), with square size proportional to the inverse-variance weight assigned in the meta-analysis. Horizontal lines indicate 95% confidence intervals (95% CI) around each study estimate. Within each subgroup, pooled prevalence estimates were calculated using a random-effects model, represented by diamonds. The prediction interval reflects the expected range of prevalence estimates in comparable future studies. The x-axis represents the estimated CPTSD prevalence proportion.

Supplementary Figure 5. Subgroup meta-analysis of complex post-traumatic stress disorder (CPTSD) prevalence by validation of the measurement tools.

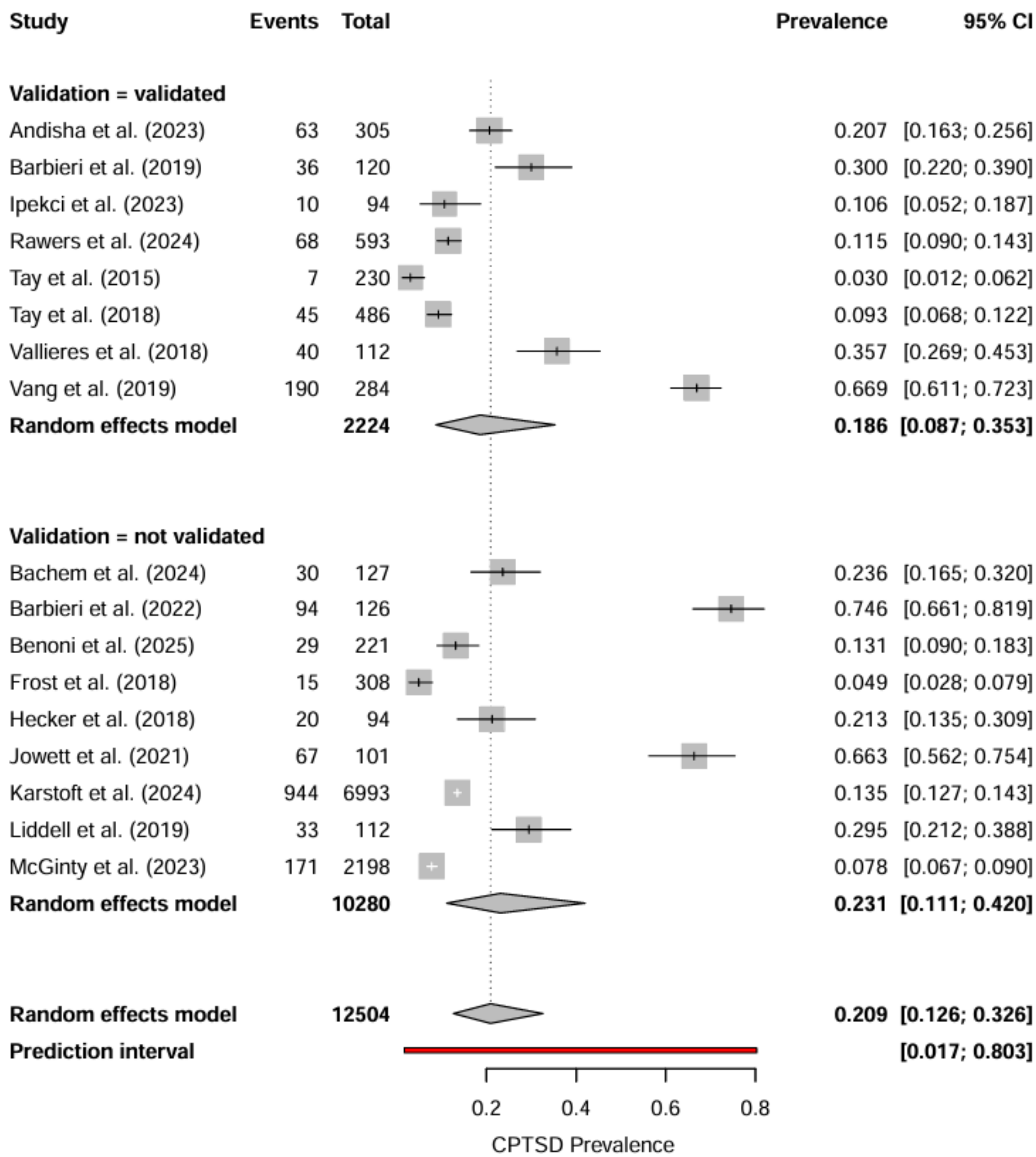

Forest plot showing the subgroup analysis of studies using an instrument validated in the language and population studied in forcibly displaced populations versus those using an instrument without such validation in this population. For each study, the square represents the estimated prevalence (proportion of participants meeting CPTSD criteria), with square size proportional to the inverse-variance weight assigned in the meta-analysis. Horizontal lines indicate 95% confidence intervals (95% CI) around each study estimate. Within each subgroup, pooled prevalence estimates were calculated using a random-effects model, represented by diamonds. The prediction interval reflects the expected range of prevalence estimates in comparable future studies. The x-axis represents the estimated CPTSD prevalence proportion.
